# Supplementary material for: Pathogenic variants in SLF2 and SMC5 cause segmented chromosomes and mosaic variegated hyperploidy
Source: Nat Commun. 2022 Nov 4;13:6664. doi: 10.1038/s41467-022-34349-8 (PMC9636423; doi:10.1038/s41467-022-34349-8)
Supplement: Supplementary file 1 — Supplementary Information [file 41467_2022_34349_MOESM1_ESM.pdf]

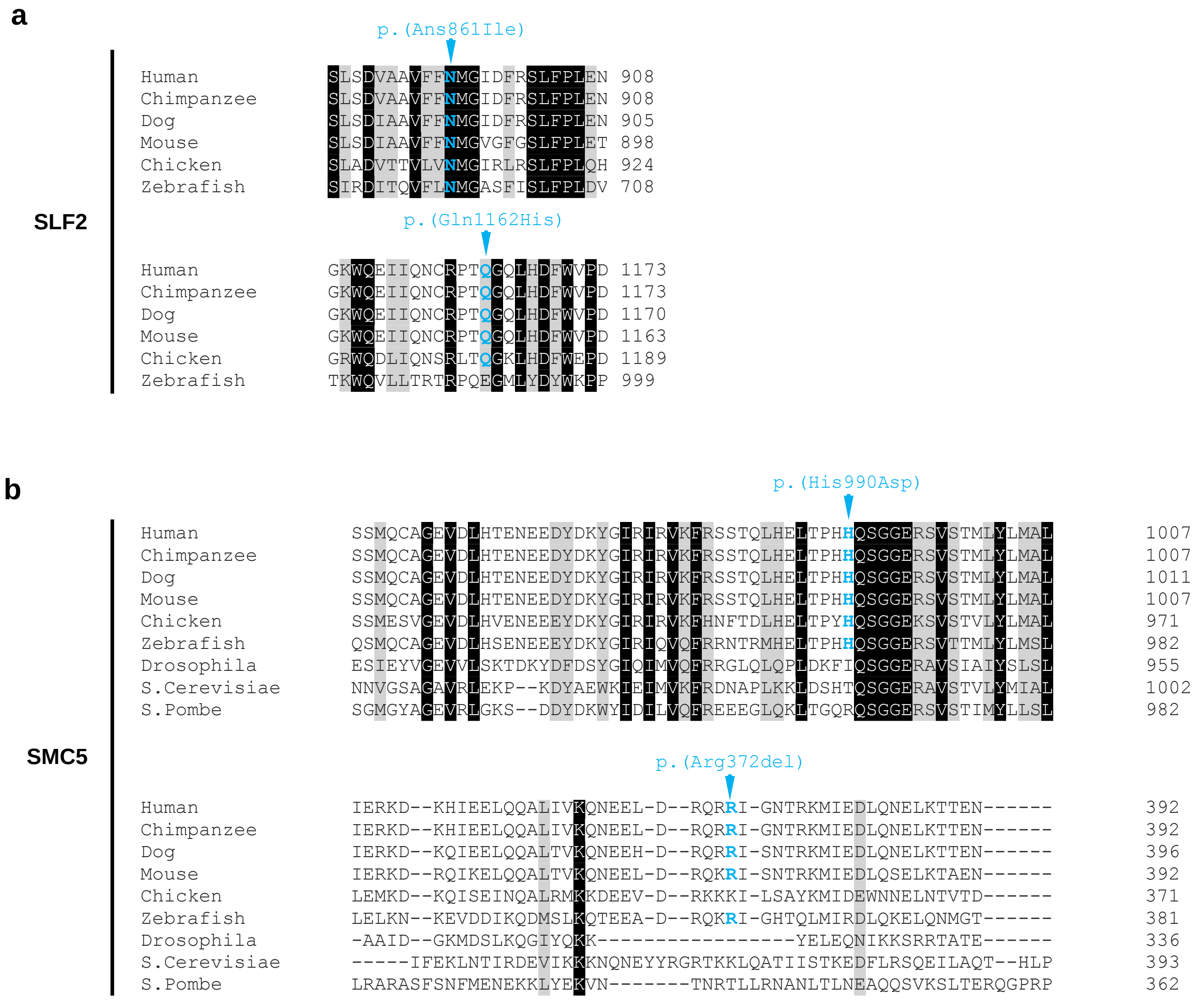

Supplementary Figure 1: Conservation of SLF2 and SMC5 amino acids mutated in patients

a Amino acid alignment of SLF2 protein from different species showing the degree of evolutionary conservation of disease causing SLF2 point variants, generated using Clustal Omega. Blue arrows indicate the missense variants present in SLF2-P2 (p.Gln1162His), and SLF2-P4-1 and SLF2-P4-2 (p.Asn861Ile). **b** Amino acid alignment of SMC5 protein from different species, generated using Clustal Omega, showing the degree of evolutionary conservation of the disease causing SMC5 point variants p.(Arg372del), present in SMC5-P7, and p.(His990Asp), present in SMC5-P8, SMC5-P9-1 and SMC5-P9-2. Blue arrows indicate location of the variants.

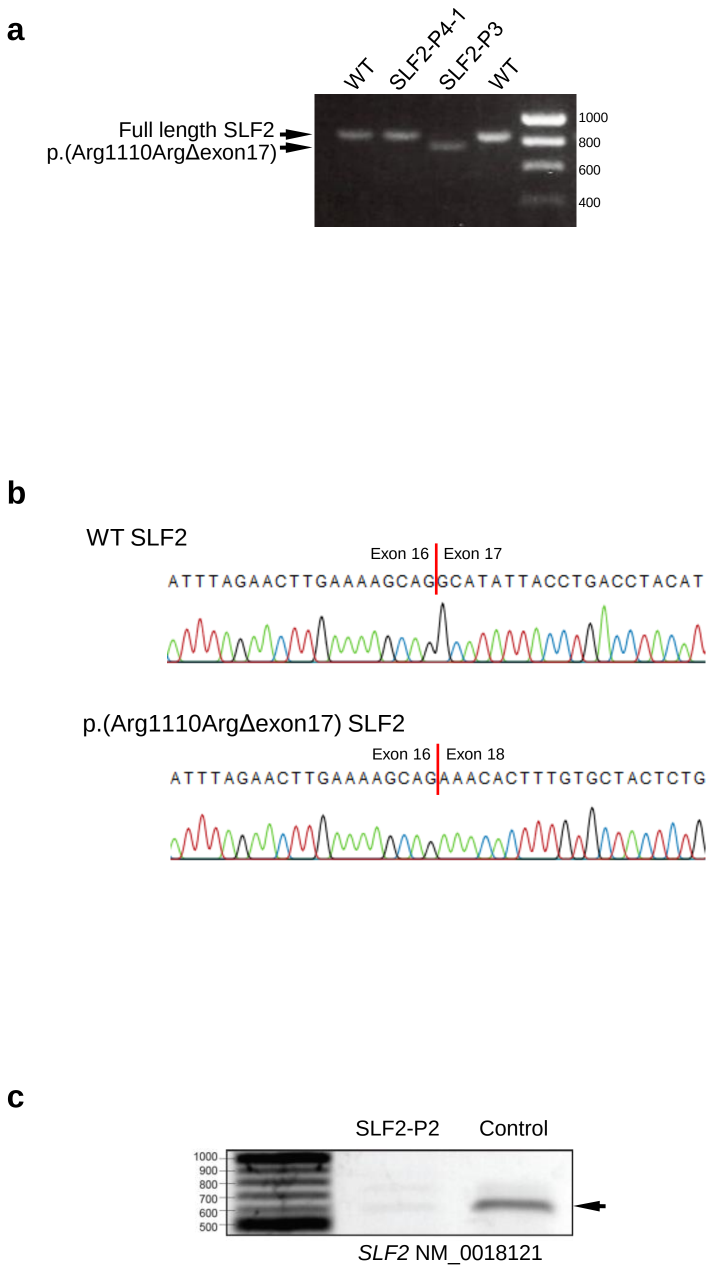

Supplementary Figure 2: Analysis of *SLF2* mRNA in SLF2-P2, SLF2-P3 and SLF2-P4-1

a PCR amplification of *SLF2* from cDNA derived from heathy normal WT individuals or SLF2 patients SLF2-P4-1 and SLF2-P3. **b** Chromatograms showing the skipping of exon 17 in the p.(Arg1110ArgΔexon17) variant from patient SLF2-P3. **c** A fragment of *SLF2* transcript (NM_018121.4) was amplified by RT-PCR from whole blood-derived mRNA from SLF2 patient SLF2-P2, as well as an age and sex matched control sample. This image is representative of three independent experiments with similar results.

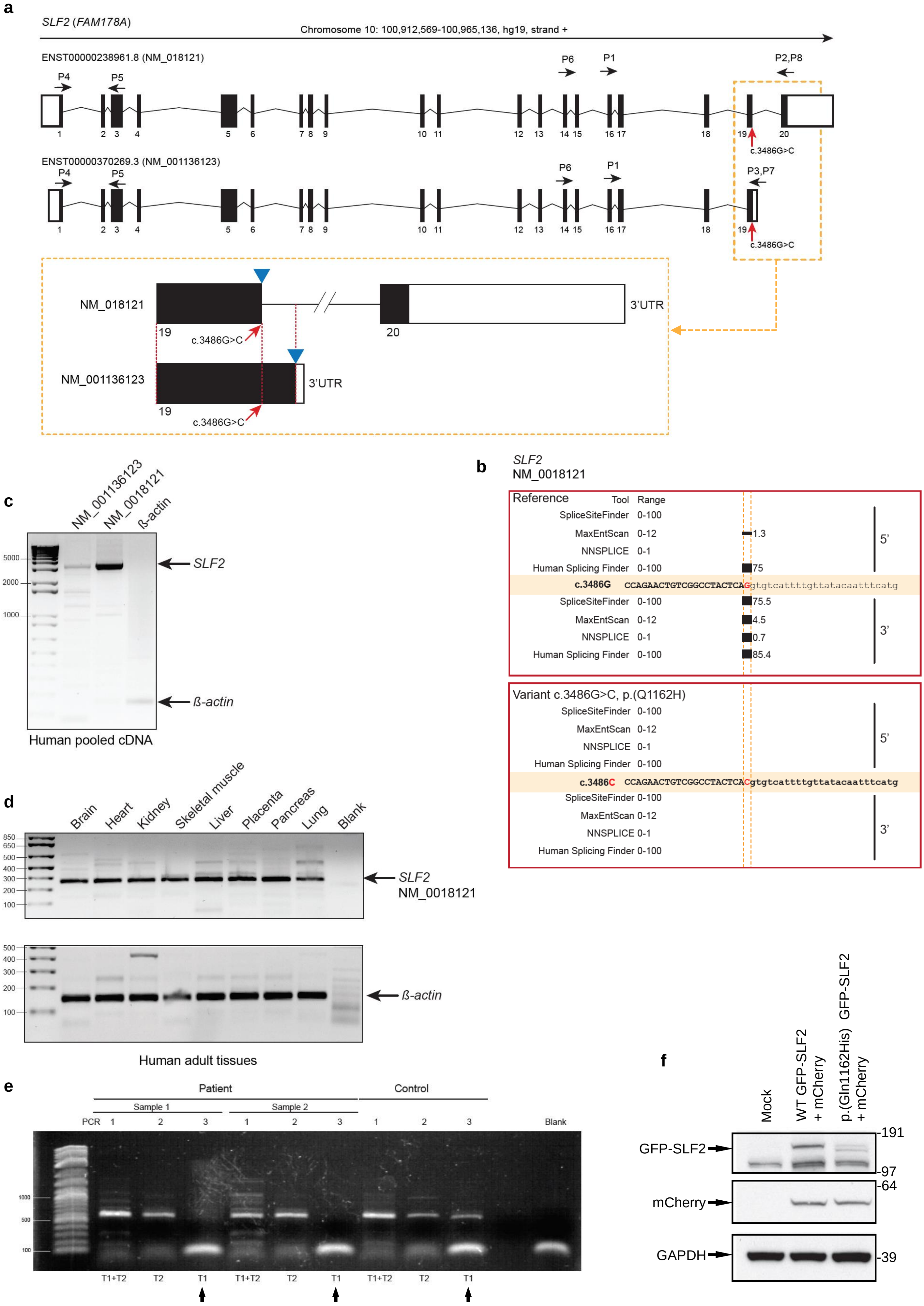
a
SLF2 (FAM178A)
Chromosome 10: 100,912,569-100,965,136, hg19, strand +
ENST00000238961.8 (NM_018121)
P4
P5
P6
P1
P2,P8
1 2 3 4 5 6 7 8 9 10 11 12 13 14 15 16 17 18 19 20
c.3486G>C
ENST00000370269.3 (NM_001136123)
P3,P7
NM_018121
19
20
3'UTR
NM_001136123
b
SLF2
NM_0018121
Reference
Tool Range
SpliceSiteFinder 0-100
MaxEntScan 0-12
NNSPLICE 0-1
Human Splicing Finder 0-100
1.3
75
c.3486G CCAGAACTGTCGGCCTACTCAGgtgtcattttgttatacaatttcatg
75.5
4.5
0.7
85.4
5'
3'
Variant c.3486G>C, p.(Q1162H)
c.3486C CCAGAACTGTCGGCCTACTCACgtgtcattttgttatacaatttcatg
c
NM_001136123
NM_0018121
ß-actin
5000
2000
1000
SLF2
Human pooled cDNA
d
Brain
Heart
Kidney
Skeletal muscle
Liver
Placenta
Pancreas
Lung
Blank
850
650
500
400
300
200
100
NM_0018121
Human adult tissues
e
Patient
Control
Sample 1
Sample 2
PCR 1 2 3 1 2 3 1 2 3
Blank
1000
500
100
T1+T2
T2
T1
f
Mock
WT GFP-SLF2 + mCherry
p.(Gln1162His) GFP-SLF2 + mCherry
GFP-SLF2
mCherry
GAPDH
-191
-97
-64
-39

Supplementary Figure 3: Analysis of *SLF2* mRNA in SLF2-P2

a Top: Schematic of the two longest annotated *SLF2* transcripts, NM_018121.4 and NM_001136123.2 containing 20 and 19 exons, respectively. Black arrows indicate position of primers used for amplification of *SLF2*. Variant c.3486G>C, (p.Gln1162His; red arrow) affects the last nucleotide of NM_018121 exon 19 (splice donor). Bottom: Enlarged view of the 3' terminal regions of the NM_018121 and NM_001136123 transcripts. Variant c.3486G>C, (p.Gln1162His) is indicated as a red arrow. Blue arrowhead shows stop codon used by either NM_018121 or NM_001136123 transcripts. Red dashed lines indicate identical sequences between NM_018121 and NM_001136123 transcripts. **b** Bioinformatic predictions indicate disrupted splicing at the exon 19 donor site (NM_018121) by the c.3486G>C, (p.Gln1162His) variant. MaxEntScan, NNSPLICE, Human Splicing Finder have been used with Alamut software to examine the probability of splicing through a donor (top, delineated by red box, labelled 5') or acceptor paradigm (bottom, labelled 3'). Range of possible values is indicated. Values obtained with each tool are indicated as black boxes. Variant c.3486G>C, p.(Gln1162His), is indicated in red. **c** RT-PCR on pooled cDNA from healthy human tissues using isoform-specific primers. Arrows show the expected size for NM_001136123 (primers P1 and P3), NM_018121 (primers P1 and P2) and *β-actin*. **d** RT-PCR of NM_018121 using cDNA derived from eight different adult tissues indicates ubiquitous expression using primers P1 and P2 (Supplementary Table S4). **e** RT-PCR on patient or control cDNA obtained from whole blood extracts indicates an isoform-specific splice defect leading to disruption of NM_018121. Primers specifically amplify NM_018121 (P6 and P8, T1, PCR3), NM_001136123 (P6 and P7, T2, PCR2) or both NM_018121 and NM_001136123 (P4 and P5, T1+T2, PCR1). Black arrows indicate result for transcript NM_018121, absent in the affected individual although present in control sample. **F** Representative immunoblot analysis of cell extracts from U-2 OS cells transiently transfected with constructs expressing either WT SLF2 or the patient associated SLF2 variant p.(Gln1162His) tagged with GFP. SLF2 constructs were mixed with an equal amount of mCherry expressing vector as a transfection control. Experiments in panels c, d, e and f are representative of three independent experiments with similar results.

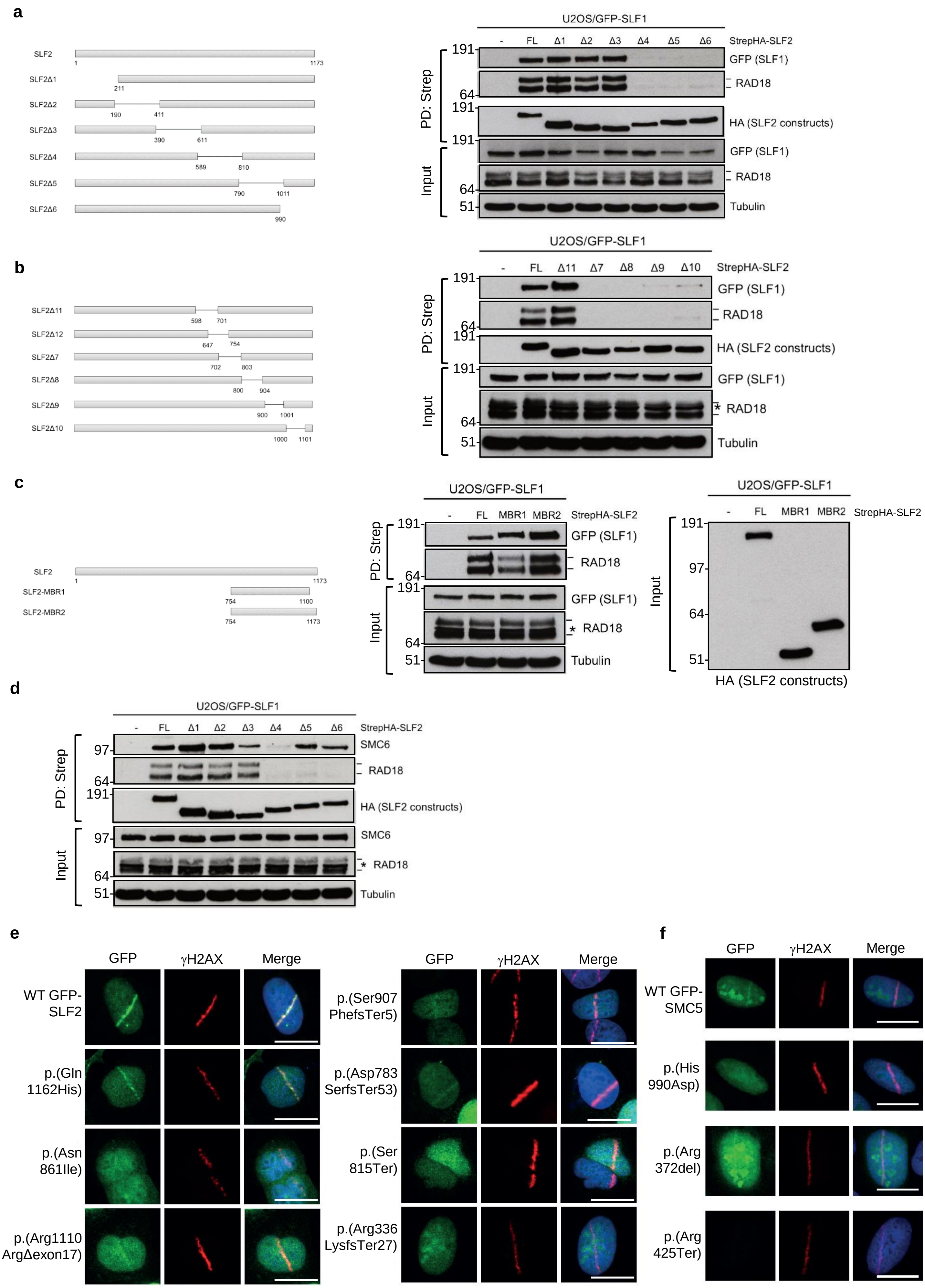

a
SLF2
SLF2Δ1
SLF2Δ2
SLF2Δ3
SLF2Δ4
SLF2Δ5
SLF2Δ6
U2OS/GFP-SLF1
- FL Δ1 Δ2 Δ3 Δ4 Δ5 Δ6 StrepHA-SLF2
PD: Strep
Input
GFP (SLF1)
RAD18
HA (SLF2 constructs)
Tubulin
b
SLF2Δ11
SLF2Δ12
SLF2Δ7
SLF2Δ8
SLF2Δ9
SLF2Δ10
U2OS/GFP-SLF1
- FL Δ11 Δ7 Δ8 Δ9 Δ10 StrepHA-SLF2
c
SLF2
SLF2-MBR1
SLF2-MBR2
- FL MBR1 MBR2 StrepHA-SLF2
HA (SLF2 constructs)
d
SMC6
e
GFP
γH2AX
Merge
WT GFP-SLF2
p.(Gln 1162His)
p.(Asn 861Ile)
p.(Arg1110 ArgΔexon17)
p.(Ser907 PhefsTer5)
p.(Asp783 SerfsTer53)
p.(Ser 815Ter)
p.(Arg336 LysfsTer27)
f
WT GFP-SMC5
p.(His 990Asp)
p.(Arg 372del)
p.(Arg 425Ter)

Supplementary Figure 4: Analysis of RAD18-SLF1/2-SMC5/6 complex interactions and recruitment to DNA damage

a & **b** Co-immunoprecipitation of SLF2 deletion mutant interacting proteins with SLF1 and RAD18. (left) Schematic of SLF2 deletion mutants. (right) U-2 OS cells transfected with SLF2 deletion constructs were subject to HA-streptavidin pulldown (DP) and immunoblotted with the indicated antibodies to determine binding of GFP-SLF1 and RAD18. **c** Co-immunoprecipitation of SLF2 minimal binding region (MBR) interacting proteins. (left) Schematic of SLF2 MBR constructs. (right) U-2 OS cells transfected with SLF2 MBR constructs were subject to HA-streptavidin pulldown and immunoblotted with the indicated antibodies. **d** Co-immunoprecipitation of SLF2 deletion mutant interacting proteins as in (a) with SMC6 and RAD18. **e** Representative images of U-2 OS cells transiently transfected with WT or mutant GFP-SLF2 constructs after laser micro-irradiation. **f** Representative images of U-2 OS cells transiently transfected with WT or mutant GFP-SMC5 constructs after laser micro-irradiation. Note that nuclear GFP signal is lost from p.(Arg425Ter) with pre-extraction. Cells in (e & f) were recovered for 1 hour post irradiation and CSK pre-extracted prior to fixation, staining and imaging (scale bar = 20 μM). Experiments in panels a, b, c, d, e and f are representative of three independent experiments with similar results.

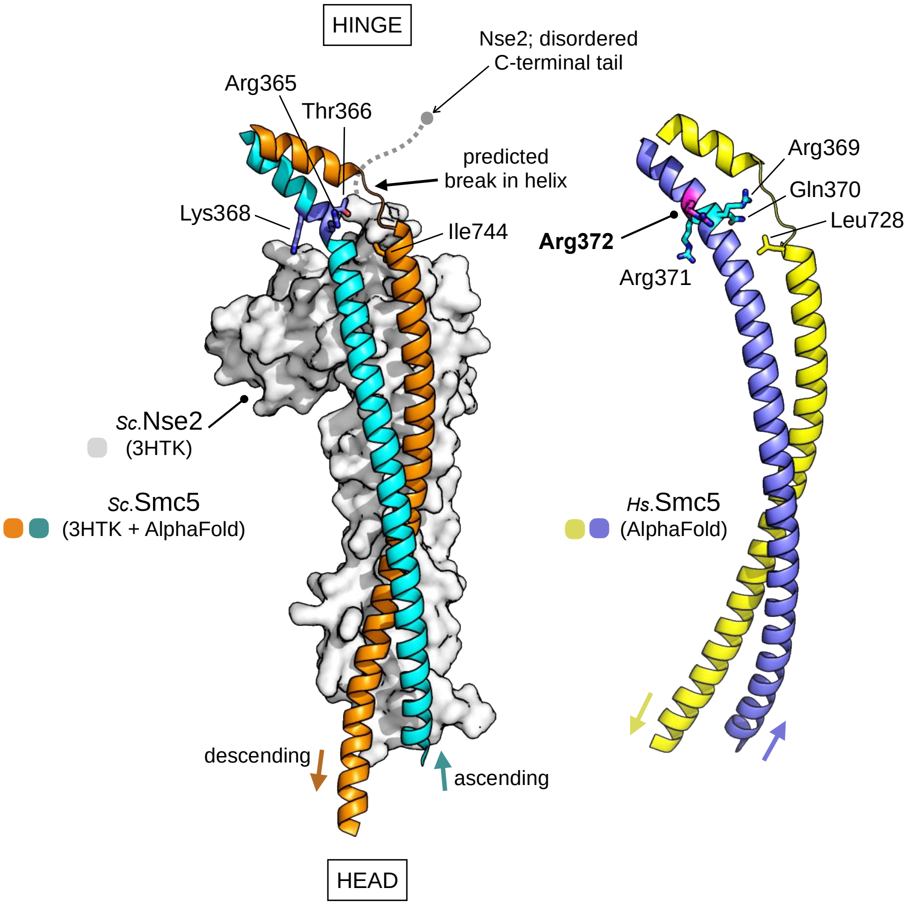

Supplementary Figure 5: Structural modelling of the SMC5 p.(Del372Arg) mutation.

(Left) Secondary structure model showing selected amino acid side chains from the X-ray crystal structure of a short section of *Saccharomyces cerevisiae* Smc5 (PDB: 3HTK) in complex with Nse2 (grey surface). An AlphaFold model for the relevant section of the Smc5 arm has been structurally superposed (AF-Q08204-F1) to extend the two helices towards the hinge (as this region is absent from the crystal structure). (Right) Secondary structure model showing the equivalent region from an AlphaFold model of human SMC5 (AF-Q8IY18-F1). Arg372 is located within a small, charged motif (369-RQRR-372) that sits near to a region of predicted disorder in the opposing helix; a similar motif can be found in budding yeast (365-RTKK-368). A hydrophobic residue (Ile744 and Leu728, in budding yeast and humans, respectively) serves to anchor the restarted (descending) helix. The directionality of each helix from the Smc5 arm (N- to C-terminus) is indicated by an arrow, heading either towards (ascending) or away (descending) from the hinge. Of note, the C-terminal tail of Nse2 emerges in close proximity to the predicted break in the descending helix.

a

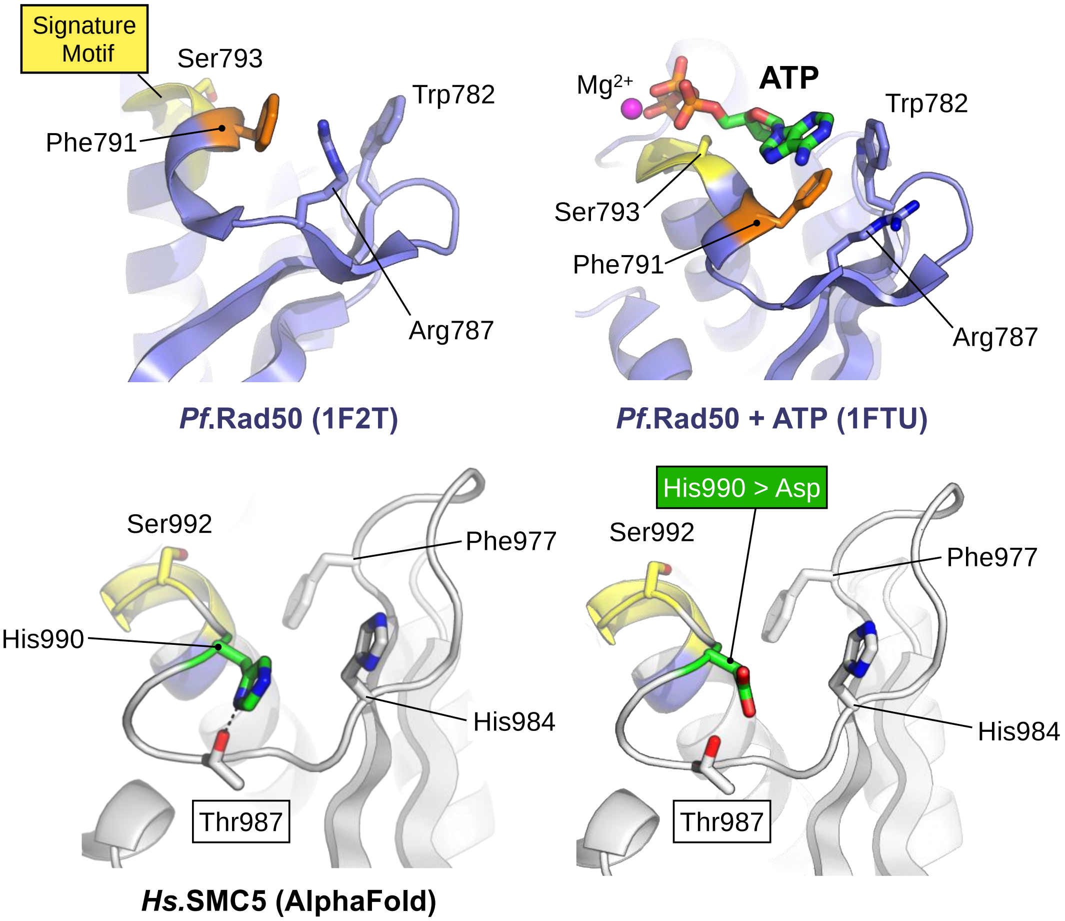

b

Analysis Program*	ΔΔG (kcal.mol^{-1})	Predicted Effect
DynaMut	0.587	Stabilising
mCSM	0.342	Stabilising
DUET	0.368	Stabilising
SDM	-0.200	Destabilising
EnCOM	0.164	Destabilising
Analysis Program	ΔΔSVib (kcal.mol^{-1}.K^{-1})	Predicted Effect
ENCoM	-2.05	Decrease of molecule flexibility

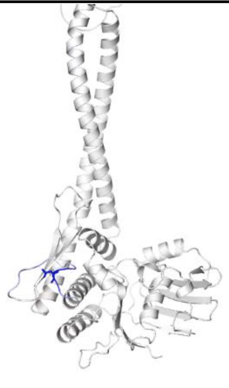

* all values obtained from DynaMut webserver

Supplementary Figure 6: Structural modelling of the SMC5 p.(His990Asp) mutation.

a (top) Secondary structure models showing selected amino acid side chains from *Pyrococcus furiosus* RAD50 (*Pf*.Rad50) in un-liganded (left) and ATP-bound forms (right); PDB accession codes 1F2T and 1FTU respectively. The ATP-binding cassette (ABC) signature motif, containing Ser793, is additionally highlighted with carbon atoms coloured in yellow. The side chains of Phe791, Arg787 and Trp782 are repositioned as a result of ATP-binding and interaction with a second Rad50 monomer (not shown). **a** (bottom) Comparative view for the same region of human SMC5 (AlphaFold model, AF-Q8IY18-F1; UniProt entry SMC_HUMAN). Side chains for amino acids in equivalent positions to those shown in the top panel are shown in stick representation. His990 of human SMC5 is structurally equivalent to Phe791 of *Pf.*Rad50 (carbon atoms coloured green and orange respectively) but is also within hydrogen-bonding distance of the side chain of Thr987 (black dotted line). Mutation of His990 to Asp (p.His990Asp) is likely to be tolerated, without any gross effects on protein folding as no major steric clashes are predicted by the change in amino acid identity [Mutagenesis Wizard, PyMOL]. However, its introduction would affect the overall charge and electronics of the region accepting the adenine moiety of bound ATP. In addition the p.(His990Asp) mutation would affect stacking/packing interactions with the side chains of both His984 and Phe977 (by analogy to *Pf*.Rad50). **b** Summary of prediction outcomes from the DynaMut webserver: http://biosig.unimelb.edu.au/dynamut. The SMC5 p.(His990Asp) patient mutation is predicted to generate only small increases or decreases in $\Delta\Delta G$ and thus no gross effect on the overall protein fold. A moderate decrease in molecule flexibility is predicted, but this is limited to just the loop containing the affected amino acid (see inset molecular model, region coloured in dark blue).

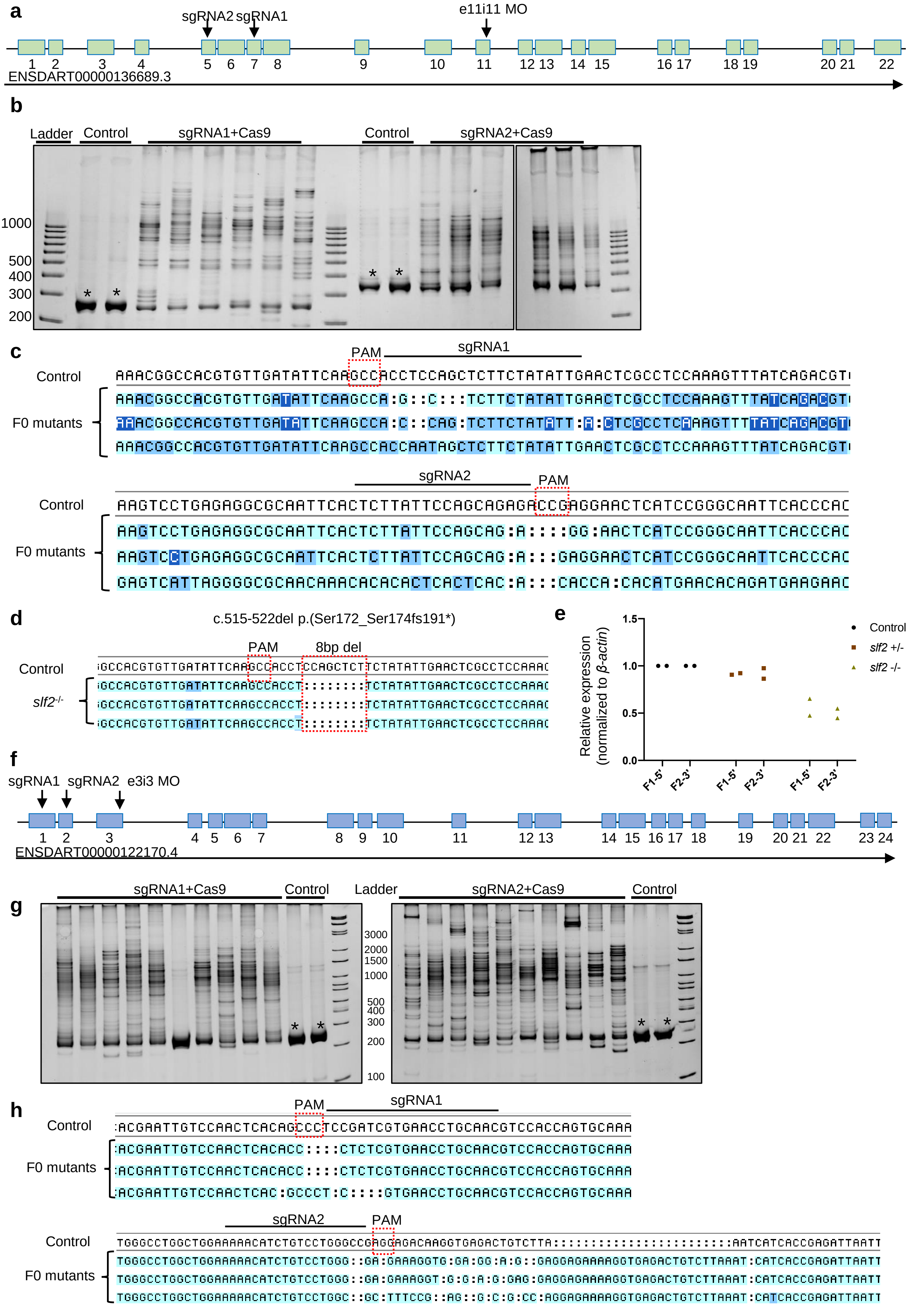
a
sgRNA2 sgRNA1
e11i11 MO
1 2 3 4 5 6 7 8 9 10 11 12 13 14 15 16 17 18 19 20 21 22
ENSDART00000136689.3
b
Ladder
Control
sgRNA1+Cas9
Control
sgRNA2+Cas9
1000
500
400
300
200
c
PAM
sgRNA1
Control
AAACGGCCACGTGTTGATATTCAAGCCACCTCCAGCTCTTCTATATTGAACTCGCCTCCAAAGTTTATCAGACGT
F0 mutants
AAACGGCCACGTGTTGATATTCAAGCCA:G::C:::TCTTCTATATTGAACTCGCCTCCAAAGTTTATCAGACGT
AAACGGCCACGTGTTGATATTCAAGCCA:C::CAG:TCTTCTATATT:A:CTCGCCTCAAAGTTTATCAGACGT
AAACGGCCACGTGTTGATATTCAAGCCACCAATAGCTCTTCTATATTGAACTCGCCTCCAAAGTTTATCAGACGT
sgRNA2
PAM
Control
AAGTCCTGAGAGGCGCAATTCACTCTTATTCCAGCAGAGACCGAGGAACTCATCCGGGCAATTCACCCAC
F0 mutants
AAGTCCTGAGAGGCGCAATTCACTCTTATTCCAGCAG:A::::GG:AACTCATCCGGGCAATTCACCCAC
AAGTCCTGAGAGGCGCAATTCACTCTTATTCCAGCAG:A:::GAGGAACTCATCCGGGCAATTCACCCAC
GAGTCATTAGGGGCGCAACAAACACACACTCACTCAC:A:::CACCA:CACATGAACACAGATGAAGAAC
d
c.515-522del p.(Ser172_Ser174fs191*)
PAM
8bp del
Control
GCCACGTGTTGATATTCAAGCCACCTCCAGCTCTTCTATATTGAACTCGCCTCCAAAC
slf2-/-
GCCACGTGTTGATATTCAAGCCACCT::::::::TCTATATTGAACTCGCCTCCAAAC
GCCACGTGTTGATATTCAAGCCACCT::::::::TCTATATTGAACTCGCCTCCAAAC
GCCACGTGTTGATATTCAAGCCACCT::::::::TCTATATTGAACTCGCCTCCAAAC
e
Relative expression (normalized to β-actin)
1.5
1.0
0.5
0.0
F1-5' F2-3' F1-5' F2-3' F1-5' F2-3'
Control
slf2 +/-
slf2 -/-
f
sgRNA1 sgRNA2 e3i3 MO
1 2 3 4 5 6 7 8 9 10 11 12 13 14 15 16 17 18 19 20 21 22 23 24
ENSDART00000122170.4
g
sgRNA1+Cas9
Control
Ladder
sgRNA2+Cas9
Control
3000
2000
1500
1000
500
400
300
200
100
h
PAM
sgRNA1
Control
ACGAATTGTCCAACTCACAGCCCTCCGATCGTGAACCTGCAACGTCCACCAGTGCAAA
F0 mutants
ACGAATTGTCCAACTCACACC::::CTCTCGTGAACCTGCAACGTCCACCAGTGCAAA
ACGAATTGTCCAACTCACACC::::CTCTCGTGAACCTGCAACGTCCACCAGTGCAAA
ACGAATTGTCCAACTCAC:GCCCT:C::::GTGAACCTGCAACGTCCACCAGTGCAAA
sgRNA2
PAM
Control
TGGGCCTGGCTGGAAAAACATCTGTCCTGGGCCGAGGAGACAAGGTGAGACTGTCTTA::::::::::::::::::::::::AATCATCACCGAGATTAATT
F0 mutants
TGGGCCTGGCTGGAAAAACATCTGTCCTGGG::GA:GAAAGGTG:GA:GG:A:G::GAGGAGAAAGGTGAGACTGTCTTAAAT:CATCACCGAGATTAATT
TGGGCCTGGCTGGAAAAACATCTGTCCTGGG::GA:GAAAGGT:G:G:A:G:GAG:GAGGAGAAAGGTGAGACTGTCTTAAAT:CATCACCGAGATTAATT
TGGGCCTGGCTGGAAAAACATCTGTCCTGGC::GC:TTTCCG::AG::G:C:G:CC:AGGAGAAAGGTGAGACTGTCTTAAAT:CATCACCGAGATTAATT

Supplementary Figure 7: Efficiency of reagents used to target *slf2* and *smc5* in zebrafish larvae.

a Schematic of the *Danio rerio slf2* genomic locus (GRCz11). Filled rectangles denote coding exons; black lines indicate introns. Target position of single guide RNAs (sgRNA) and morpholinos (MO) used are indicated with vertical arrows. **b** Polyacrylamide gel image showing heteroduplex analysis of PCR products amplified from genomic DNA harvested at 2 dpf from control embryos and embryos injected with *slf2* sgRNAs plus Cas9 protein. Embryos revealed high mosaicism of frameshifting insertions and deletions at each respective target site (*slf2* sgRNA1: 82%; *slf2* sgRNA2: 70%). Asterisks indicate homoduplexes of WT PCR product. **c** Representative sequences generated from PCR products cloned into TOPO-TA vectors. Plasmids were purified from individual colonies and subjected to direct sequencing, revealing insertions and deletions in *slf2* F0 mutant larvae. Protospacer adjacent motif (PAM) is shown with red dashed box. **d** Representative sequences confirming an 8 bp deletion in *slf2*$^{-/-}$ mutants. **e** qRT-PCR depicts 50% reduction in *slf2* mRNA level normalized to *β-actin*. F1-5' and F2-3' indicate two different primer sets complementary to the 5' and 3' regions of the *slf2* mRNA, respectively. n=2 independent experiments. **f** Schematic of the Danio *rerio smc5* genomic locus (GRCz11). Filled rectangles denote coding exons; black lines denote introns. Target position of single guide (sg) RNAs and MO used are indicated with vertical arrows. **g** Polyacrylamide gel image showing heteroduplex analysis of PCR products amplified from genomic DNA harvested at 2 dpf from control embryos and embryos injected with *smc5* sgRNA plus Cas9 protein. High mosaicism of frameshifting insertions and deletions at each respective target site is visible (*smc5* sgRNA1: 92%; *smc5* sgRNA2: 80%). Asterisks indicate homoduplexes of WT PCR product. **h** Representative sequences generated from PCR products (panel g) cloned into TOPO-TA vectors. Plasmids were purified from individual colonies and subjected to direct sequencing, revealing insertions and deletions in *smc5* F0 mutant larvae. Protospacer adjacent motif (PAM) is shown with red dashed box. Polyacrylamide gels in panels b and g were generated for screening purposes and are representative of one experimental repeat.

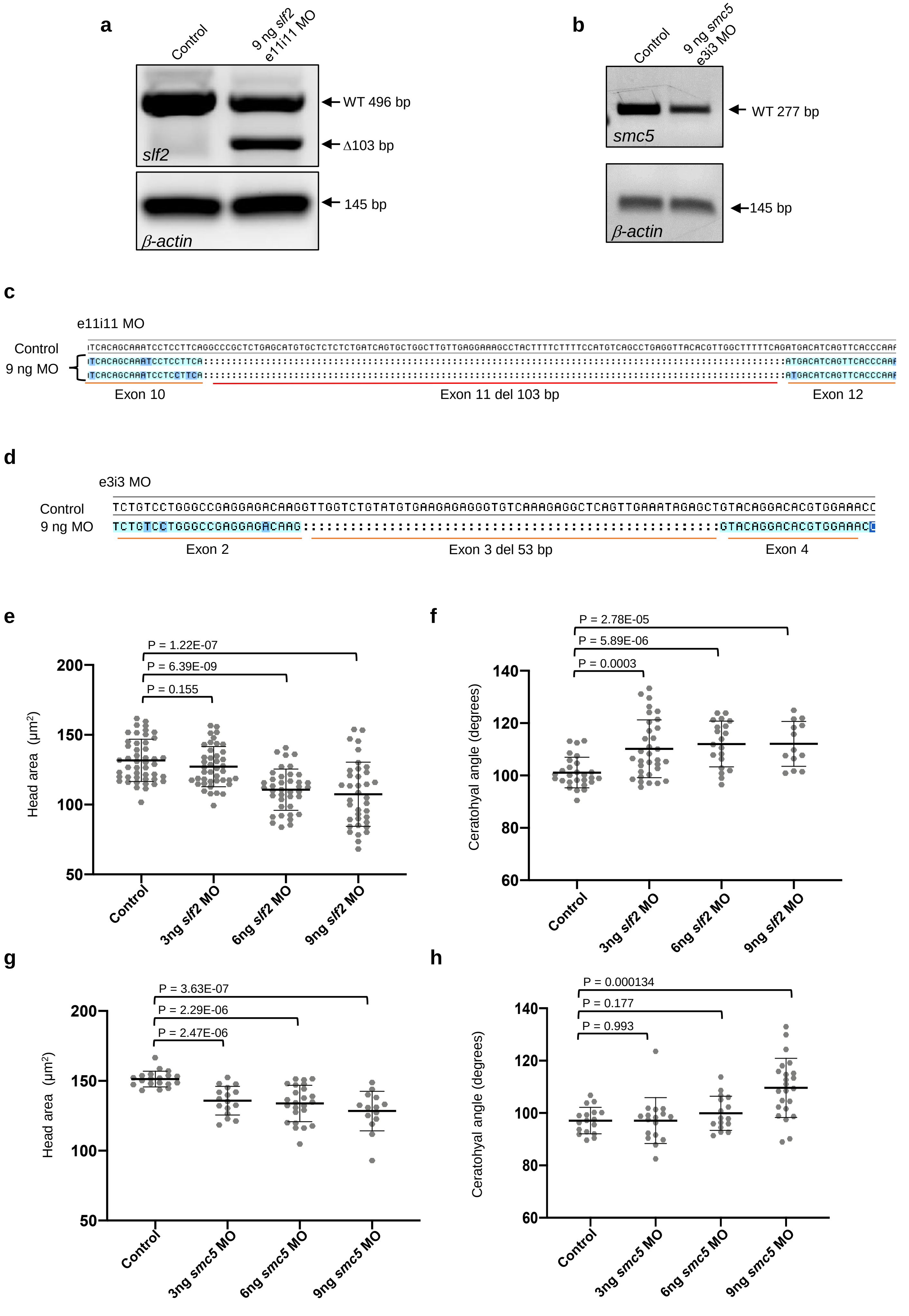
a
Control
9 ng slf2 e11i11 MO
WT 496 bp
Δ103 bp
slf2
145 bp
β-actin
b
Control
9 ng smc5 e3i3 MO
WT 277 bp
smc5
145 bp
β-actin
c
e11i11 MO
Control
9 ng MO
Exon 10
Exon 11 del 103 bp
Exon 12
d
e3i3 MO
Control
9 ng MO
Exon 2
Exon 3 del 53 bp
Exon 4
e
P = 1.22E-07
P = 6.39E-09
P = 0.155
Head area (μm²)
Control
3ng slf2 MO
6ng slf2 MO
9ng slf2 MO
f
P = 2.78E-05
P = 5.89E-06
P = 0.0003
Ceratohyal angle (degrees)
Control
3ng slf2 MO
6ng slf2 MO
9ng slf2 MO
g
P = 3.63E-07
P = 2.29E-06
P = 2.47E-06
Head area (μm²)
Control
3ng smc5 MO
6ng smc5 MO
9ng smc5 MO
h
P = 0.000134
P = 0.177
P = 0.993
Ceratohyal angle (degrees)
Control
3ng smc5 MO
6ng smc5 MO
9ng smc5 MO

Supplementary Figure 8: Loss of *slf2* and *smc5* in zebrafish give rise to microcephaly and aberrant craniofacial patterning

a Agarose gel images show exon exclusion of *slf2* exon 11 in morphants (MO) resulting in a 103 bp deletion (Δ) as determined by RT-PCR and sequencing. **b** Agarose gel images show semi-quantitative reduction of WT message in *smc5* MO as determined by RT-PCR. Agarose gels in b and c are representative of one experimental repeat. **c** RT-PCR product sequence confirmation of exon 11 skipping in MO as determined by cloning and sequencing of the lower *slf2* band in the morphant lane of (a). **d** RT-PCR sequence confirmation of exon 3 skipping in MO as determined by cloning and sequencing the *smc5* band in the morphant lane of (b). **e-h** Quantification of lateral head size (e & g) (left to right; 47, 42, 39, 37 embryos/condition were analysed from 3 independent experiments for panel e, and 27, 33, 20, 13 embryos/condition were analysed from 3 independent experiments for panel g), and ceratohyal angle measurements (f & h) (left to right; 19, 16, 24, 13 embryos/condition were analysed from 3 independent experiments for panel f, and 17, 16, 16, 24 embryos/condition were analysed from 3 independent experiments for panel h), of larvae injected with different doses (3 ng, 6 ng and 9 ng) of MO, or MO with co-injection of human WT *SLF2* or *SMC5* mRNA. Error bars represent standard deviation of the mean. Statistical differences were determined with an unpaired Student's t-test (two sided).

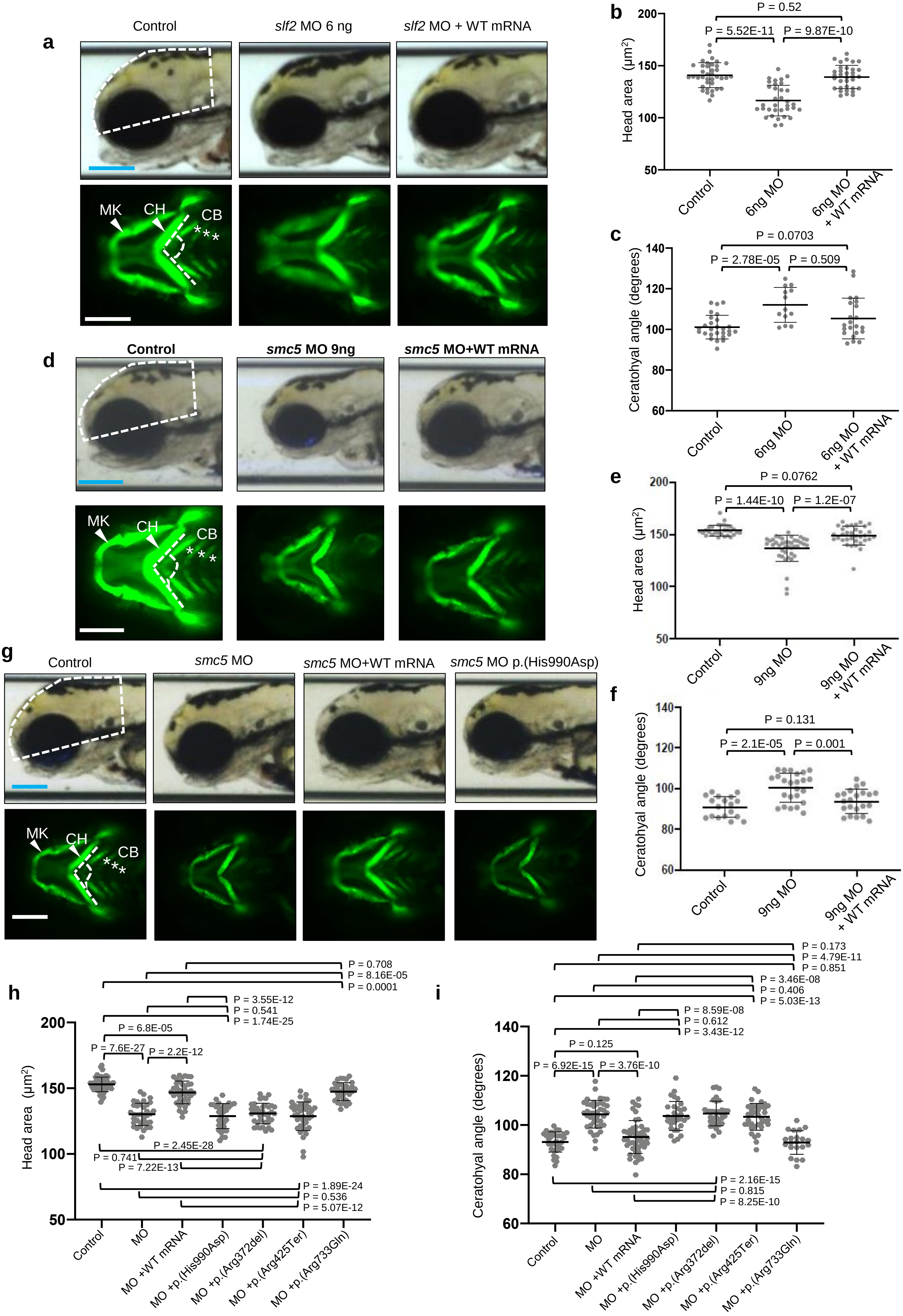

a
Control
slf2 MO 6 ng
slf2 MO + WT mRNA
MK
CH
CB
b
P = 0.52
P = 5.52E-11
P = 9.87E-10
Head area (μm²)
Control
6ng MO
6ng MO + WT mRNA
c
P = 0.0703
P = 2.78E-05
P = 0.509
Ceratohyal angle (degrees)
d
Control
smc5 MO 9ng
smc5 MO+WT mRNA
e
P = 0.0762
P = 1.44E-10
P = 1.2E-07
9ng MO
9ng MO + WT mRNA
f
P = 0.131
P = 2.1E-05
P = 0.001
g
smc5 MO
smc5 MO+WT mRNA
smc5 MO p.(His990Asp)
h
P = 0.708
P = 8.16E-05
P = 0.0001
P = 3.55E-12
P = 0.541
P = 1.74E-25
P = 6.8E-05
P = 7.6E-27
P = 2.2E-12
P = 2.45E-28
P = 0.741
P = 7.22E-13
P = 1.89E-24
P = 0.536
P = 5.07E-12
MO
MO +WT mRNA
MO +p.(His990Asp)
MO +p.(Arg372del)
MO +p.(Arg425Ter)
MO +p.(Arg733Gln)
i
P = 0.173
P = 4.79E-11
P = 0.851
P = 3.46E-08
P = 0.406
P = 5.03E-13
P = 8.59E-08
P = 0.612
P = 3.43E-12
P = 0.125
P = 6.92E-15
P = 3.76E-10
P = 2.16E-15
P = 0.815
P = 8.25E-10

Supplementary Figure 9: Loss of *slf2* and *smc5* in zebrafish give rise to microcephaly and aberrant craniofacial patterning.

a Representative bright field lateral (top) and ventral images of the GFP signal in the anterior region of *-1.4col1a1:egfp* transgenic reporter larvae (bottom) showing controls, *slf2* morphants (MO) and *slf2* MO rescued with human WT *SLF2* mRNA, respectively. **b** & **c** Quantification of lateral head size (b) (left to right; 38, 34, 34 embryos/condition from 3 independent experiments) or ceratohyal angle measurements (c) (left to right; 27, 13, 22 embryos/condition from 3 independent experiments). **d** Representative bright field lateral (top) and ventral images of the GFP signal in the anterior region of *-1.4col1a1:egfp* transgenic reporter larvae (bottom) showing controls, *smc5* MO and *smc5* MO rescued with human WT *SMC5* mRNA, respectively. **e** & **f** Quantification of lateral head size (e) (left to right; 46, 45, 45 embryos/condition from 3 independent experiments) or ceratohyal angle measurements (f) (left to right; 18, 24, 22 embryos/condition from 3 independent experiments). **g** Top, representative lateral bright field images; and bottom, representative ventral GFP signal showing in the mandible of *-1.4col1a1:egfp* transgenic reporter larvae at 3 dpf. Images show head size (top) and craniofacial patterning (bottom) in controls, in *smc5* MO, MO rescued with human SMC5 WT mRNA, and MO complemented with p.(His990Asp) patient variant. Top, white dashed shape depicts head size measured; botoom, white dashed lines show the ceratohyal angle. Abbreviations: MK, Meckel's cartilage; CH, ceratohyal cartilage (indicated with arrowheads, respectively); CB, ceratobranchial arches (asterisks); MO, morpholino. Scale bar, 300 μm, with equivalent sizing across panels. **h** & **i** Quantification of lateral head size (h) (left to right; 54, 36, 39, 33, 39, 40, 34 embryos/condition from 3 independent experiments) and ceratohyal angle measurements (i) (left to right; 34, 42, 49, 32, 33, 37, 21 embryos/condition from 3 independent experiments) of larvae injected with MO alone, co-injection of MO with human WT or variant encoding mRNA; p.(Arg733Gln) is a negative control (rs59648118; 16 homozygotes in gnomAD). For all panels: Statistical differences were determined with an unpaired Student's t-test (two sided). Error bars represent standard deviation of the mean. Scale bars, 300 µm with equivalent sizing across panels.

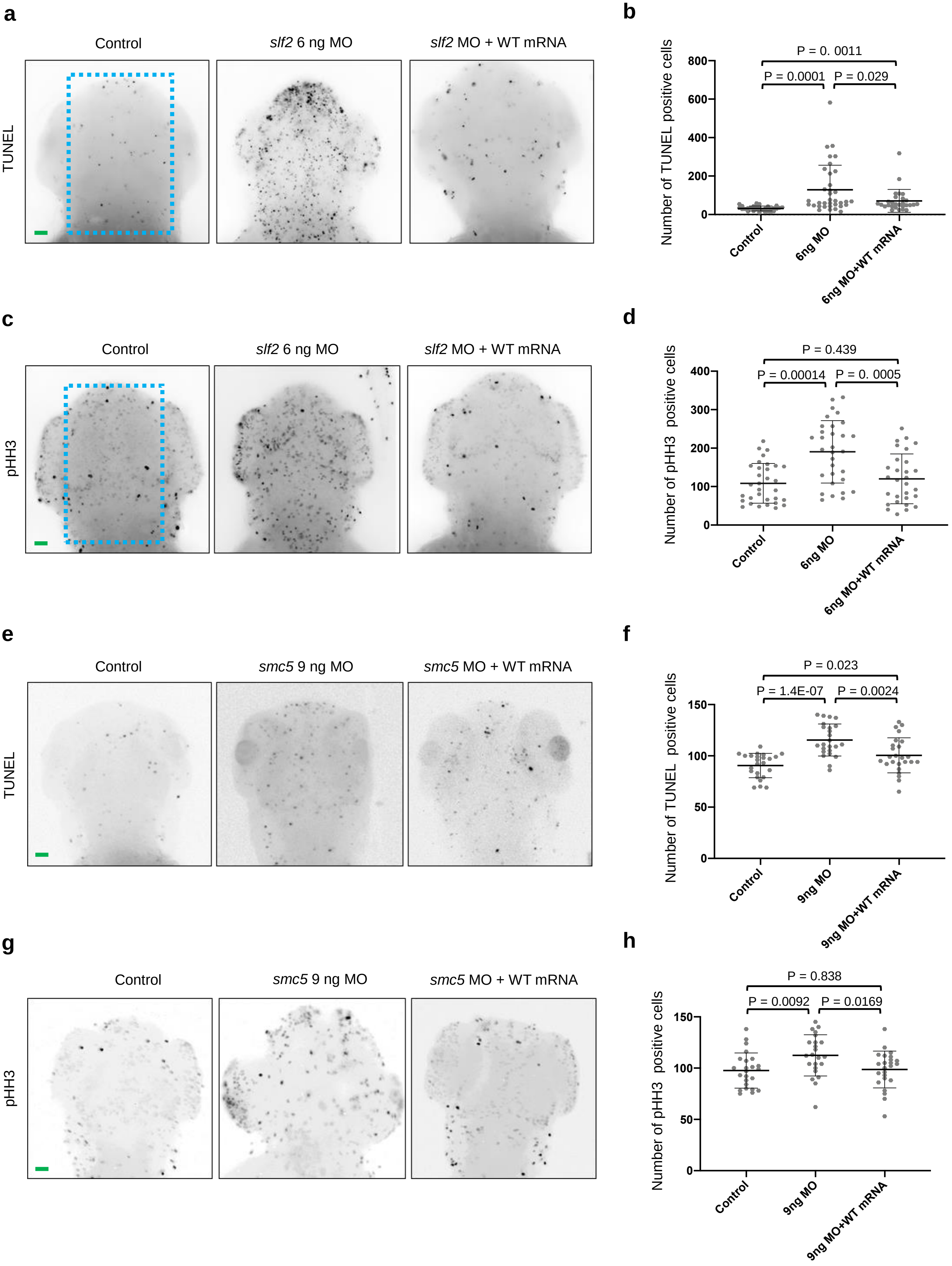
a
Control
slf2 6 ng MO
slf2 MO + WT mRNA
TUNEL
b
Number of TUNEL positive cells
800
600
400
200
0
P = 0. 0011
P = 0.0001
P = 0.029
Control
6ng MO
6ng MO+WT mRNA
c
Control
slf2 6 ng MO
slf2 MO + WT mRNA
pHH3
d
Number of pHH3 positive cells
400
300
200
100
0
P = 0.439
P = 0.00014
P = 0. 0005
Control
6ng MO
6ng MO+WT mRNA
e
Control
smc5 9 ng MO
smc5 MO + WT mRNA
TUNEL
f
Number of TUNEL positive cells
150
100
50
0
P = 0.023
P = 1.4E-07
P = 0.0024
Control
9ng MO
9ng MO+WT mRNA
g
Control
smc5 9 ng MO
smc5 MO + WT mRNA
pHH3
h
Number of pHH3 positive cells
150
100
50
0
P = 0.838
P = 0.0092
P = 0.0169
Control
9ng MO
9ng MO+WT mRNA

Supplementary Figure 10: *slf2* and *smc5* depletion induces apoptosis and altered cell cycle progression in zebrafish larvae.

a Representative dorsal inverted fluorescent images indicating TUNEL positive cells in *slf2* MO at 2 dpf. The blue dashed box indicates the region of interest (ROI). **b** Quantification of TUNEL positive cells in controls and larvae injected with *slf2* MO with or without WT mRNA (left to right; 35, 29, 28 embryos/condition from 3 independent experiments). ROI used is shown in panel (a). **c** Representative dorsal inverted fluorescent images indicating pHH3 positive cells in *slf2* MO at 2 dpf. **d** Quantification of pHH3 positive cells in larvae injected with *slf2* MO with or without WT mRNA (left to right; 24, 25, 25 embryos/condition from 3 independent experiments). ROI used was the same as that shown in panel (c). **e** Representative dorsal inverted fluorescent images show TUNEL positive cells in *smc5* MO at 3 dpf. **f** Quantification of TUNEL positive cells in controls and larvae injected with *smc5* MO with or without WT mRNA (left to right; 32, 31, 29 embryos/condition from 3 independent experiments). **g** Representative dorsal inverted fluorescent images indicating pHH3 positive cells in *smc5* MO at 2 dpf. **h** Quantification of pHH3 positive cells in controls and larvae injected with *smc5* MO and WT mRNA (left to right; 24, 23, 24 embryos/condition from 3 independent experiments). In all cases, embryos of the same developmental stage and similar magnification were assessed for all *slf2* or *smc5* conditions. Fluorescent staining in the ROI was quantified using the ImageJ (NIH) ICTN plugin. Error bars represent standard deviation of the mean. Scale bar in panels a, c, e, g: 30 μm with equivalent sizing across panels. In all cases, statistical differences were determined with an unpaired Student's t-test (two sided).

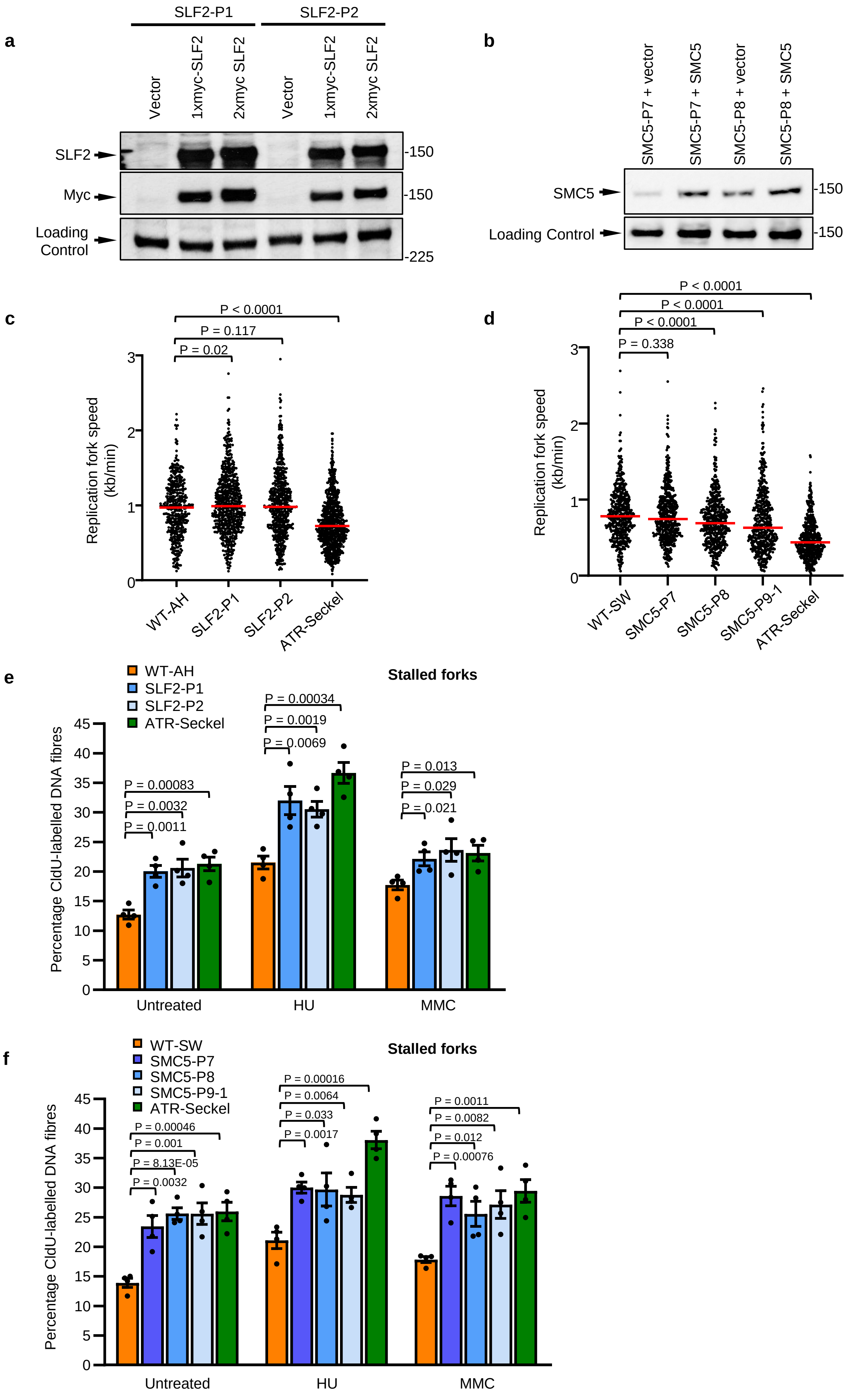

a
SLF2-P1
SLF2-P2
Vector
1xmyc-SLF2
2xmyc SLF2
Vector
1xmyc-SLF2
2xmyc SLF2
SLF2
Myc
Loading Control
-150
-150
-225
b
SMC5-P7 + vector
SMC5-P7 + SMC5
SMC5-P8 + vector
SMC5-P8 + SMC5
SMC5
Loading Control
-150
-150
c
Replication fork speed (kb/min)
P < 0.0001
P = 0.117
P = 0.02
WT-AH
SLF2-P1
SLF2-P2
ATR-Seckel
d
Replication fork speed (kb/min)
P < 0.0001
P < 0.0001
P < 0.0001
P = 0.338
WT-SW
SMC5-P7
SMC5-P8
SMC5-P9-1
ATR-Seckel
e
WT-AH
SLF2-P1
SLF2-P2
ATR-Seckel
Stalled forks
Percentage CldU-labelled DNA fibres
P = 0.00083
P = 0.0032
P = 0.0011
P = 0.00034
P = 0.0019
P = 0.0069
P = 0.013
P = 0.029
P = 0.021
Untreated
HU
MMC
f
WT-SW
SMC5-P7
SMC5-P8
SMC5-P9-1
ATR-Seckel
Stalled forks
Percentage CldU-labelled DNA fibres
P = 0.00046
P = 0.001
P = 8.13E-05
P = 0.0032
P = 0.00016
P = 0.0064
P = 0.033
P = 0.0017
P = 0.0011
P = 0.0082
P = 0.012
P = 0.00076
Untreated
HU
MMC

Supplementary Figure 11: Replication fork analysis of SLF2 and SMC5 patient-derived cell lines

a Representative immunoblot analysis of myc-SLF2 expression in SLF2 patient fibroblasts infected with lentiviruses encoding myc-tagged WT SLF2 or an empty vector. A nonspecific cross-reactive protein was used as a loading control. **b** Representative immunoblot analysis of SMC5 expression in SMC5 fibroblasts infected with lentiviruses encoding WT SMC5 or an empty vector. A nonspecific cross-reactive protein was used as a loading control. Immunoblotting analysis in panels a and b are representative of two independent experiments with similar results. **c** & **d** Replication fork velocity of ongoing forks in WT cells, SLF2 patient LCLs (c) or SMC5 patient LCLs (d). n=3 independent experiments. A minimum of 430 fork structures were counted. Red lines denote median values. A Mann-Whitney rank sum test was performed for statistical analysis. **e** & **f** DNA fibre analysis in untreated cells and cells exposed to replication stress in SLF2 patient-derived LCLs (e) or SMC5 patient-derived LCLs (f) was carried out. In untreated cells, the indicated cell lines were pulse-labelled with CldU for 20 min, and then pulse-labelled with IdU, for 20 min. For DNA fibres following MMC treatment, cells were incubated with 50 ng/ml MMC for 24 h prior to pulse-labelling with CldU and IdU. For DNA fibres following hydroxyurea (HU) treatment, cells were pulsed with CldU for 20 min, exposed to 2 mM HU for 2 h and then pulsed with IdU for 20 min. The percentage of stalled forks was quantified. n=4 independent experiments. A minimum of 650 fork structures were counted. A Student's t-test (two-sided, equal variance) was performed for statistical analysis. Error bars denote SEM.

a

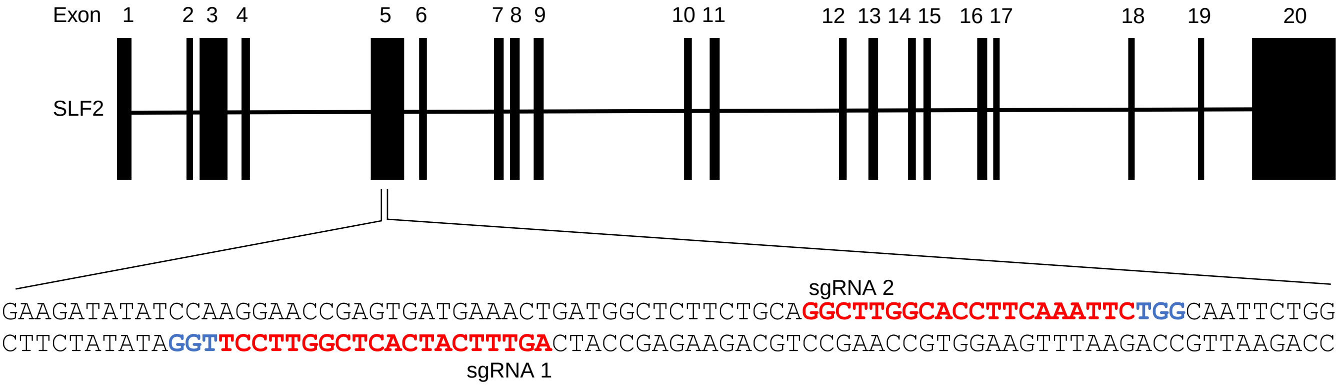

b

Clone #	Mutation
Cl. 1	c. 1192_1210dupGATGAAACTGATGGCTCTT, p.(Ser403Ter)
	c.1184_1232dupAACCGAGTGATGAAACTGATGGCTCTTCTGCAGGCTTGGCACCTTCAAA, p.(Asn411Lysfs3Ter)
	c.1185_1232dupACCGAGTGATGAAACTGATGGCTCTTCTGCAGGCTTGGCACCTTCAAA, p.(Ser410_Asn411insKPSDETDGSSAGLAPS)
Cl. 2	c.1207_1223delTCTTCTGCAGGCTTGGC, p.(Ser403Thrfs14Ter)
	c.1188_1208delGAGTGATGAAACTGATGGCTC, p.(Asp398_Ser404del)

c

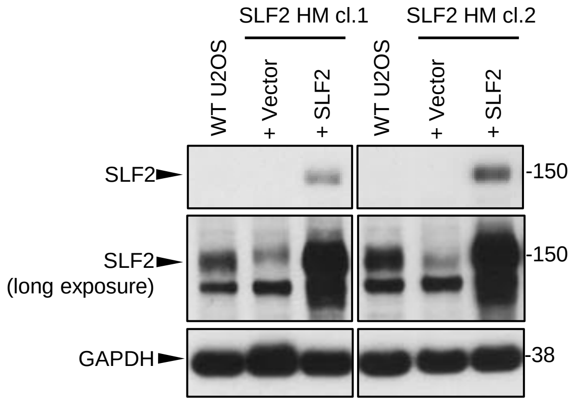

d

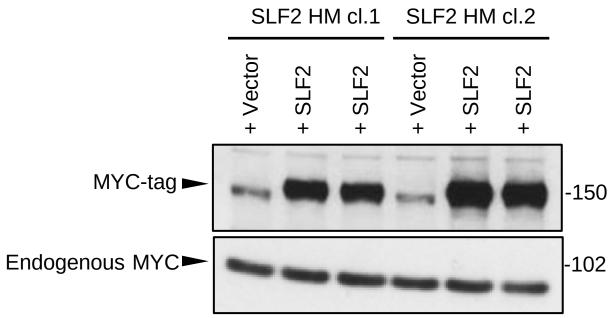

Supplementary Figure 12: Generation of U-2 OS SLF2 CRISPR hypomorphic cell lines

a Schematic of the human *SLF2* genomic locus. Filled rectangles indicate coding exons; black lines denote introns. Positions of single guide RNAs (sgRNA) are highlighted by red text and the location of the protospacer adjacent motif (PAM) is indicated by blue text. **b** Table detailing SLF2 variants present in U-2 OS SLF2 CRISPR hypomorphic (HM) clones cl.1 and cl.2. **c** Representative immunoblot analysis of SLF2 expression in U-2 OS SLF2 CRISPR HM cell lines infected with lentiviruses encoding myc-tagged WT SLF2 or an empty vector. GAPDH was used as a loading control. **d** Representative immunoblot analysis of myc-SLF2 expression in U-2 OS SLF2 CRISPR HM cell lines infected with lentiviruses encoding myc-tagged WT SLF2 or an empty vector. Endogenous c-Myc was used as a loading control. Immunoblotting analysis in panels c and d are representative of two independent experiments with similar results.

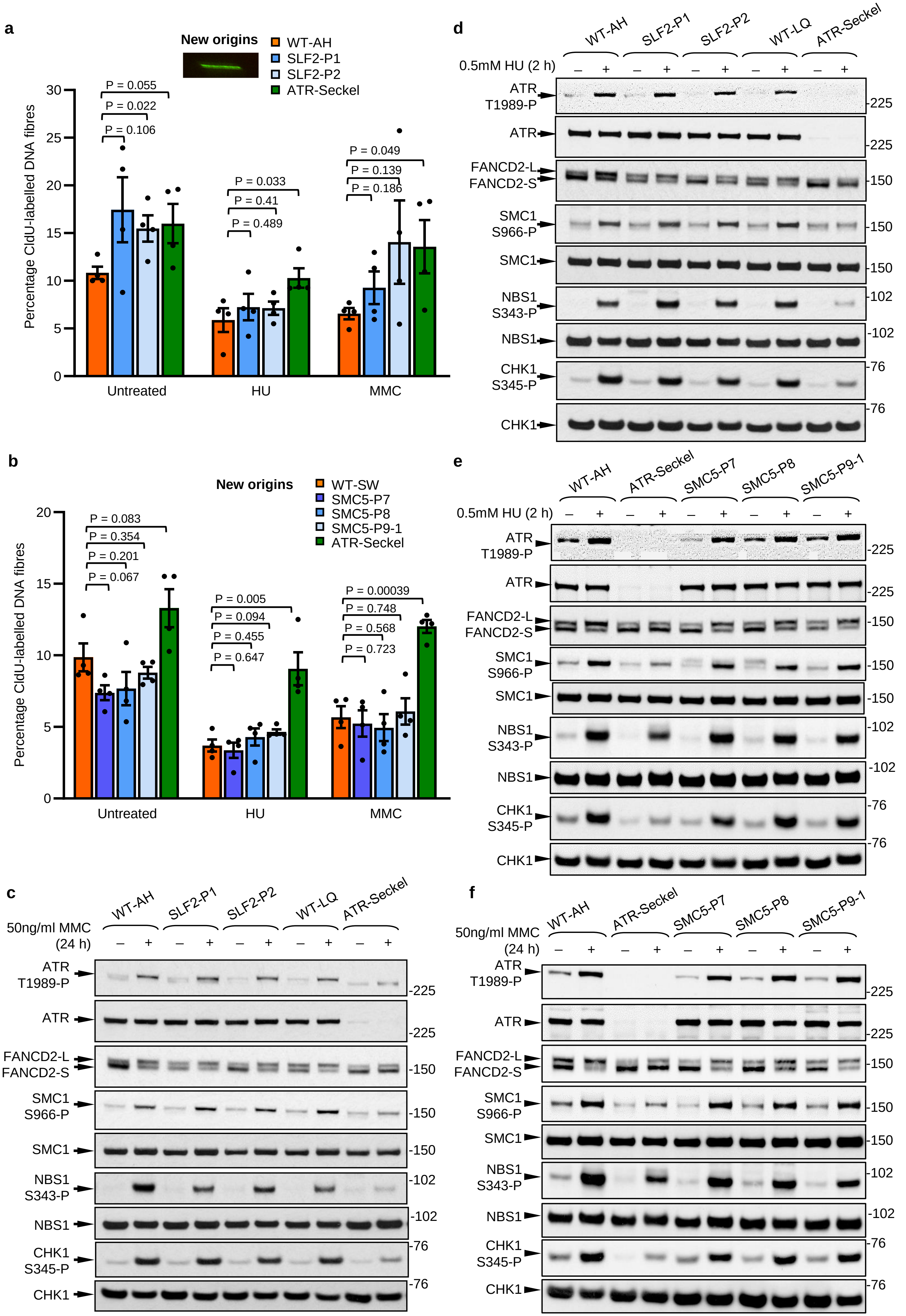
a
New origins
WT-AH
SLF2-P1
SLF2-P2
ATR-Seckel
Percentage CldU-labelled DNA fibres
30
25
20
15
10
5
0
P = 0.055
P = 0.022
P = 0.106
P = 0.033
P = 0.41
P = 0.489
P = 0.049
P = 0.139
P = 0.186
Untreated
HU
MMC
b
New origins
WT-SW
SMC5-P7
SMC5-P8
SMC5-P9-1
ATR-Seckel
Percentage CldU-labelled DNA fibres
20
15
10
5
0
P = 0.083
P = 0.354
P = 0.201
P = 0.067
P = 0.005
P = 0.094
P = 0.455
P = 0.647
P = 0.00039
P = 0.748
P = 0.568
P = 0.723
Untreated
HU
MMC
c
WT-AH
SLF2-P1
SLF2-P2
WT-LQ
ATR-Seckel
50ng/ml MMC (24 h)
– + – + – + – + – +
ATR T1989-P
ATR
FANCD2-L
FANCD2-S
SMC1 S966-P
SMC1
NBS1 S343-P
NBS1
CHK1 S345-P
CHK1
-225
-225
-150
-150
-150
-102
-102
-76
-76
d
WT-AH
SLF2-P1
SLF2-P2
WT-LQ
ATR-Seckel
0.5mM HU (2 h)
– + – + – + – + – +
ATR T1989-P
ATR
FANCD2-L
FANCD2-S
SMC1 S966-P
SMC1
NBS1 S343-P
NBS1
CHK1 S345-P
CHK1
-225
-225
-150
-150
-150
-102
-102
-76
-76
e
WT-AH
ATR-Seckel
SMC5-P7
SMC5-P8
SMC5-P9-1
0.5mM HU (2 h)
– + – + – + – + – +
ATR T1989-P
ATR
FANCD2-L
FANCD2-S
SMC1 S966-P
SMC1
NBS1 S343-P
NBS1
CHK1 S345-P
CHK1
-225
-225
-150
-150
-150
-102
-102
-76
-76
f
WT-AH
ATR-Seckel
SMC5-P7
SMC5-P8
SMC5-P9-1
50ng/ml MMC (24 h)
– + – + – + – + – +
ATR T1989-P
ATR
FANCD2-L
FANCD2-S
SMC1 S966-P
SMC1
NBS1 S343-P
NBS1
CHK1 S345-P
CHK1
-225
-225
-150
-150
-150
-102
-102
-76
-76

Supplementary Figure 13: Analysis of the ATR-CHK1 dependent replication stress response in SLF2 and SMC5 patient-derived LCLs

a & **b** DNA fibre analysis of SLF2 (a) and SMC5 (b) patient-derived LCLs was carried out as in (Supplementary Figure 11 e & f) and the percentage of new origins (IdU only) were quantified. A representative image is included. n=4 independent experiments. A minimum of 650 fork structures were counted. A Student's t-test (two-sided, equal variance) was performed for statistical analysis. Error bars denote SEM. **c-f** Representative immunoblot analysis for the indicated proteins in whole-cell extracts from SLF2 (c & d) or SMC5 (e & f) patient-derived LCLs subjected to treatment with 0.5 mM HU for 2 h (d & e) or 50 ng/ml MMC for 24 h (c & f). In all cases, immunoblotting analysis are representative of two independent experiments with similar results.

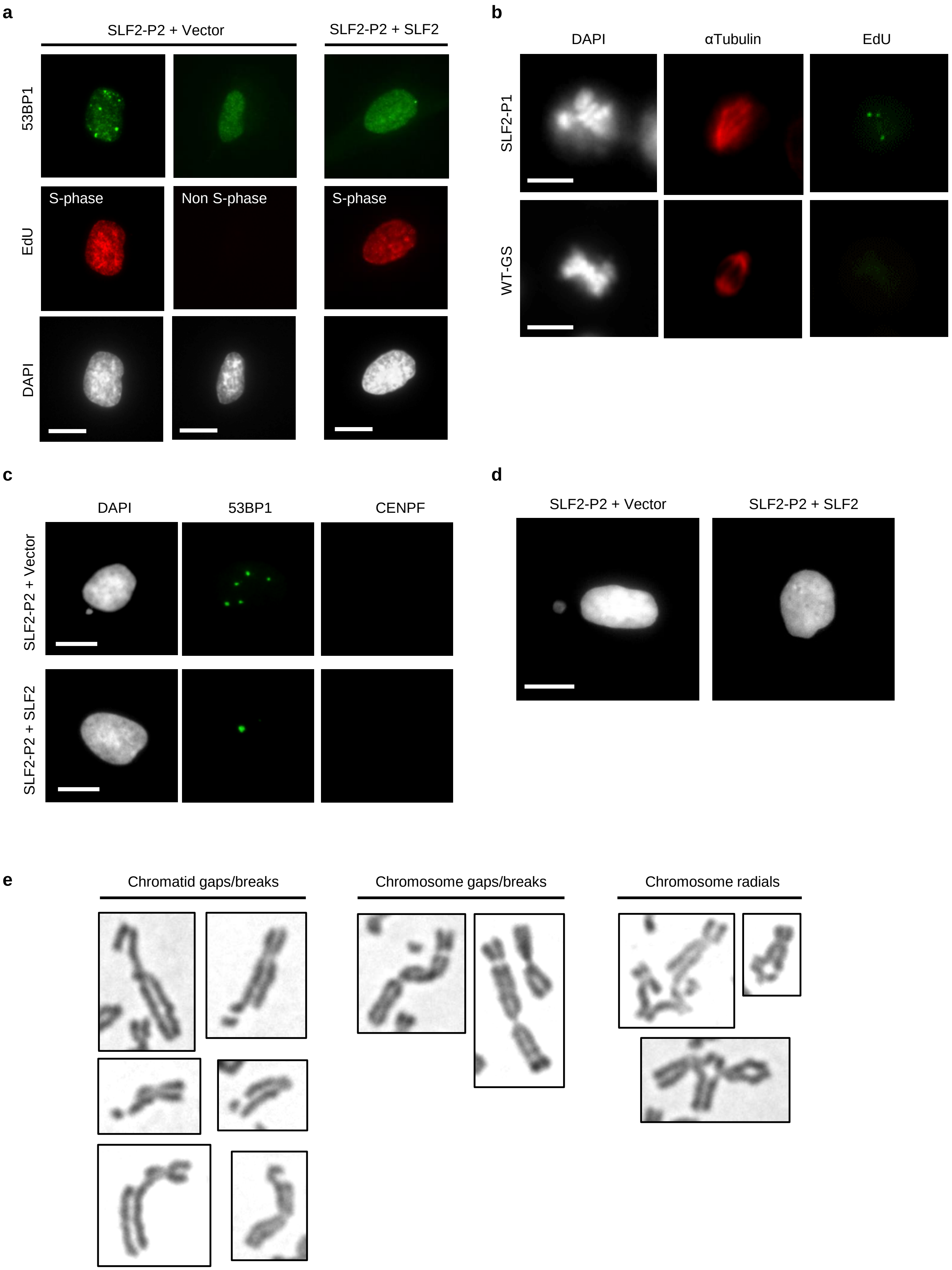
a
SLF2-P2 + Vector
SLF2-P2 + SLF2
53BP1
EdU
DAPI
S-phase
Non S-phase
S-phase
b
DAPI
αTubulin
EdU
SLF2-P1
WT-GS
c
DAPI
53BP1
CENPF
SLF2-P2 + Vector
SLF2-P2 + SLF2
d
SLF2-P2 + Vector
SLF2-P2 + SLF2
e
Chromatid gaps/breaks
Chromosome gaps/breaks
Chromosome radials

Supplementary Figure 14: Representative microscopy images of SLF2/SMC5 mutant cell lines exhibiting elevated levels of S-phase associated DNA damage

a Representative immunofluorescence microscopy images of EdU positive S-phase cells with 53BP1 foci quantified in Figure 6a. **b** Representative immunofluorescence microscopy images of mitotic cells with MiDAS quantified in Figure 6b. **c** Representative immunofluorescence microscopy images of 53BP1 bodies in CENPF negative G1 cells quantified in Figure 6c. **d** Representative immunofluorescence microscopy images of cells with micronuclei quantified in Figure 6d. **e** Representative brightfield microscopy images of different types of chromosomal aberrations quantified in Figure 6f-I, Figure 8e, Supplementary Figure S15a-b, Supplementary Figure 19d, Supplementary Figure S20c-d. In all cases, scale bars = 10 µM.

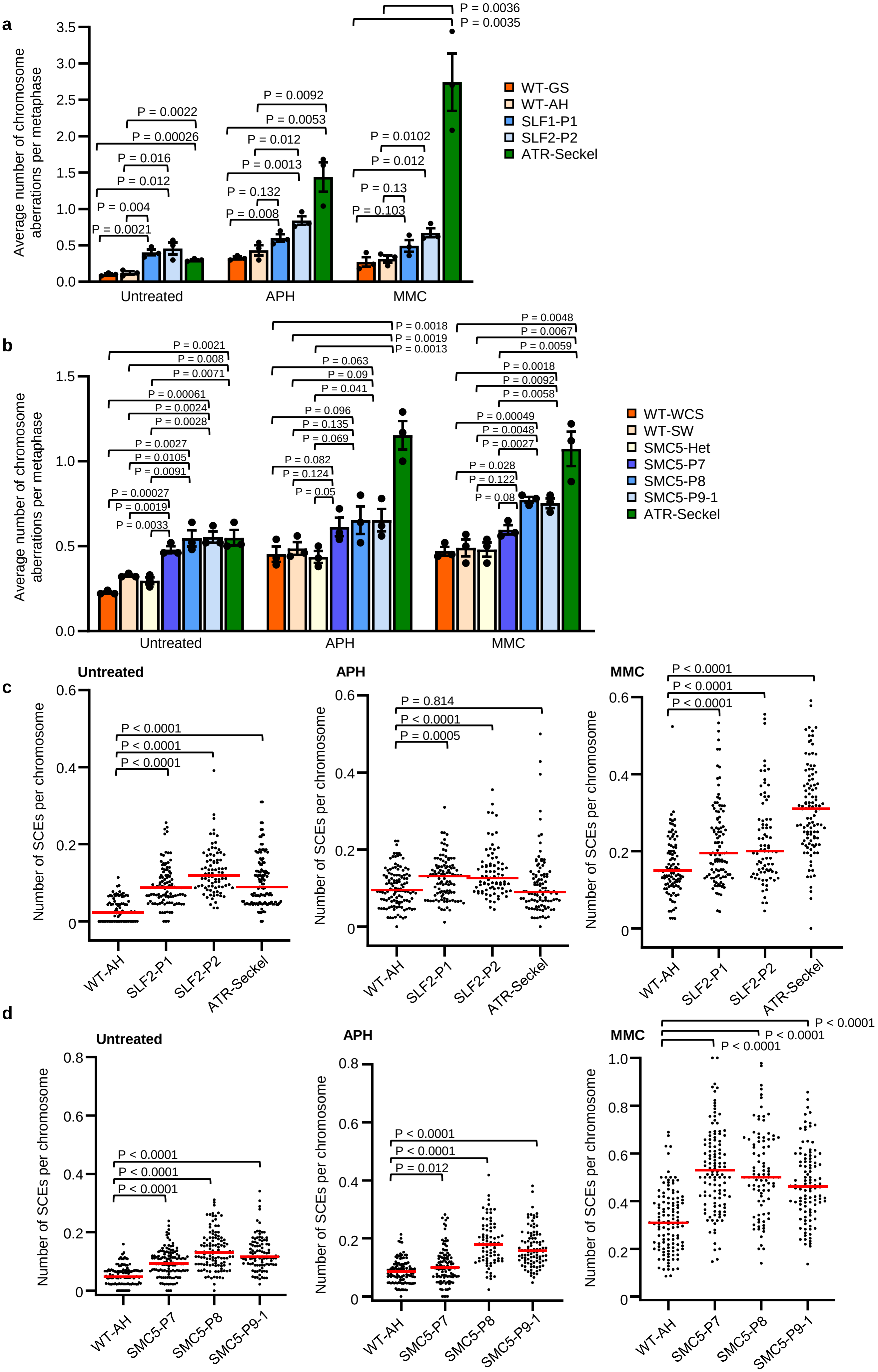
a
Average number of chromosome aberrations per metaphase
0.0
0.5
1.0
1.5
2.0
2.5
3.0
3.5
Untreated
APH
MMC
WT-GS
WT-AH
SLF1-P1
SLF2-P2
ATR-Seckel
P = 0.0021
P = 0.004
P = 0.012
P = 0.016
P = 0.00026
P = 0.0022
P = 0.008
P = 0.132
P = 0.0013
P = 0.012
P = 0.0053
P = 0.0092
P = 0.103
P = 0.13
P = 0.012
P = 0.0102
P = 0.0036
P = 0.0035
b
Average number of chromosome aberrations per metaphase
0.0
0.5
1.0
1.5
Untreated
APH
MMC
WT-WCS
WT-SW
SMC5-Het
SMC5-P7
SMC5-P8
SMC5-P9-1
ATR-Seckel
P = 0.0021
P = 0.008
P = 0.0071
P = 0.00061
P = 0.0024
P = 0.0028
P = 0.0027
P = 0.0105
P = 0.0091
P = 0.00027
P = 0.0019
P = 0.0033
P = 0.0018
P = 0.0019
P = 0.0013
P = 0.063
P = 0.09
P = 0.041
P = 0.096
P = 0.135
P = 0.069
P = 0.082
P = 0.124
P = 0.05
P = 0.0048
P = 0.0067
P = 0.0059
P = 0.0018
P = 0.0092
P = 0.0058
P = 0.00049
P = 0.0048
P = 0.0027
P = 0.028
P = 0.122
P = 0.08
c
Untreated
APH
MMC
Number of SCEs per chromosome
0
0.2
0.4
0.6
P < 0.0001
P < 0.0001
P < 0.0001
P = 0.814
P < 0.0001
P = 0.0005
P < 0.0001
P < 0.0001
P < 0.0001
WT-AH
SLF2-P1
SLF2-P2
ATR-Seckel
d
Untreated
APH
MMC
Number of SCEs per chromosome
0
0.2
0.4
0.6
0.8
1.0
P < 0.0001
P < 0.0001
P < 0.0001
P < 0.0001
P < 0.0001
P = 0.012
P < 0.0001
P < 0.0001
P < 0.0001
WT-AH
SMC5-P7
SMC5-P8
SMC5-P9-1

Supplementary Figure 15: Genome instability in SLF2/SMC5 mutant cell lines is not exacerbated by exogenous replication stress

a & **b** Quantification of the average number of chromosomal aberrations (which includes chromatid/chromosome gaps, breaks, fragments and radials) in metaphase spreads from SLF2 (a) and SMC5 (b) patient derived LCLs before treatment or following exposure to 500 nM APH or 50 ng/ml MMC for 24 h. n=3 independent experiments. A minimum of 140 metaphases were counted. A Student's t-test (two-sided, equal variance) was performed for statistical analysis. Error bars denote SEM. **c** & **d** Quantification of the average numbers of sister chromatid exchanges in metaphase spreads from SLF2 (c) and SMC5 (d) patient derived LCLs treated as in (a & b). n=3 independent experiments. A minimum of 100 metaphases were counted. Red lines denote median values. A Mann-Whitney rank sum test was performed for statistical analysis.

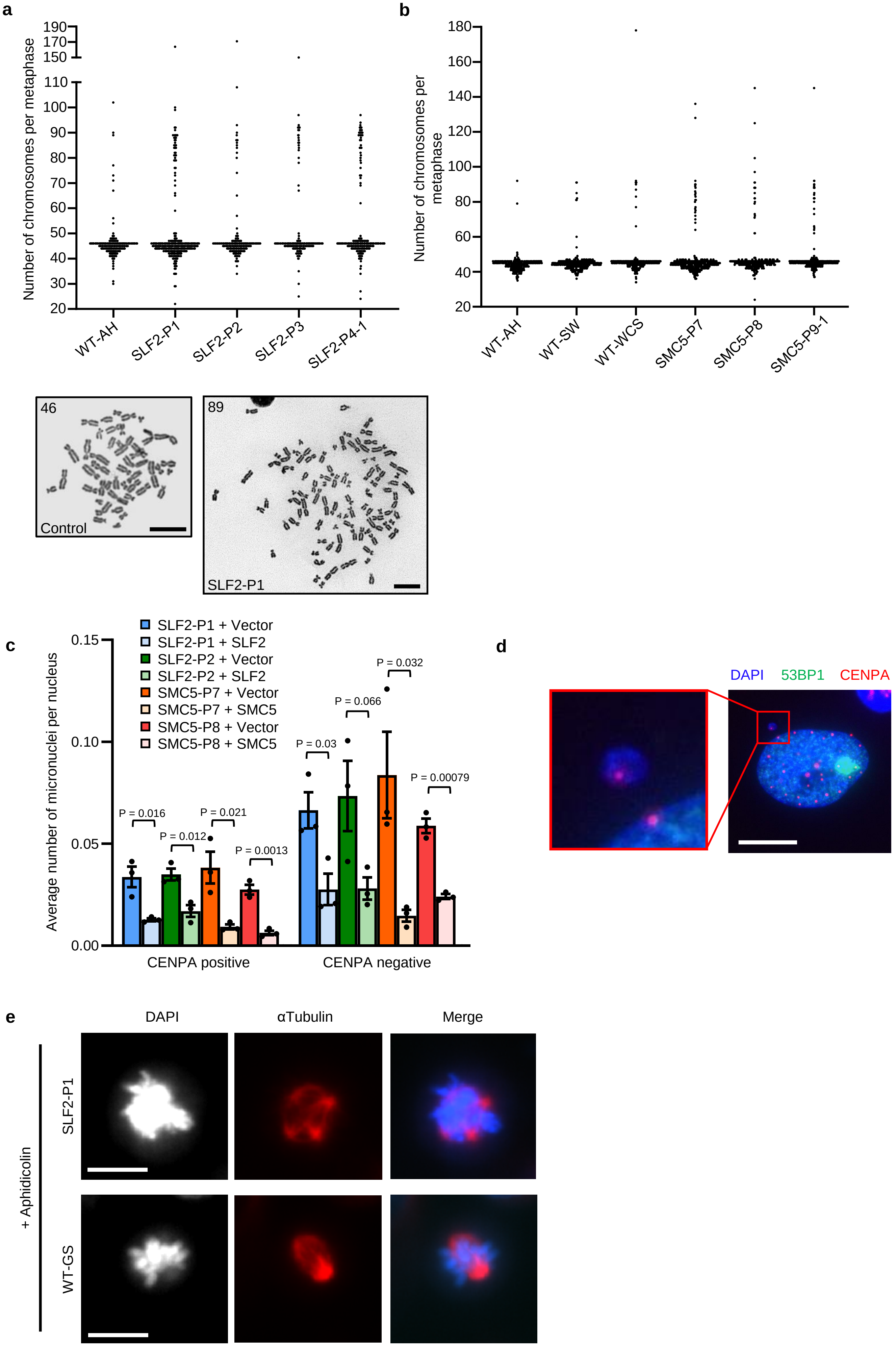
a
Number of chromosomes per metaphase
190
170
150
110
100
90
80
70
60
50
40
30
20
WT-AH
SLF2-P1
SLF2-P2
SLF2-P3
SLF2-P4-1
b
Number of chromosomes per metaphase
180
160
140
120
100
80
60
40
20
WT-AH
WT-SW
WT-WCS
SMC5-P7
SMC5-P8
SMC5-P9-1
46
Control
89
SLF2-P1
c
Average number of micronuclei per nucleus
0.15
0.10
0.05
0.00
SLF2-P1 + Vector
SLF2-P1 + SLF2
SLF2-P2 + Vector
SLF2-P2 + SLF2
SMC5-P7 + Vector
SMC5-P7 + SMC5
SMC5-P8 + Vector
SMC5-P8 + SMC5
P = 0.016
P = 0.012
P = 0.021
P = 0.0013
P = 0.03
P = 0.066
P = 0.032
P = 0.00079
CENPA positive
CENPA negative
d
DAPI
53BP1
CENPA
e
DAPI
αTubulin
Merge
+ Aphidicolin
SLF2-P1
WT-GS

Supplementary Figure 16: Levels of mosaic variegated hyperploidy in SLF2/SMC5 mutant LCLs

a Quantification of the number of chromosomes per metaphase in SLF2 patient-derived LCLs. n=3 independent experiments. A total of 300 metaphases were counted. **b** Quantification of the number of chromosomes per metaphase in SMC5 patient-derived LCLs. n=3 independent experiments. A total of 300 metaphases were counted. **c** Quantification of the average number of CENPA positive and CENPA negative micronuclei in SLF2 and SMC5 mutant fibroblast cell lines infected with lentiviruses encoding WT SLF2, WT SMC5, or an empty vector. n=3 independent experiments. A minimum of 185 micronuclei were counted. Student's t-test (two-sided, equal variance) was performed for statistical analysis. Error bars denote SEM. **d** Representative images of CENPA positive micronuclei**. e** Representative immunofluorescence microscopy images of mitotic cells from SLF2-P1 LCLs with multi-polar spindles quantified in Figure 7h. In all cases, scale bars = 10 µM.

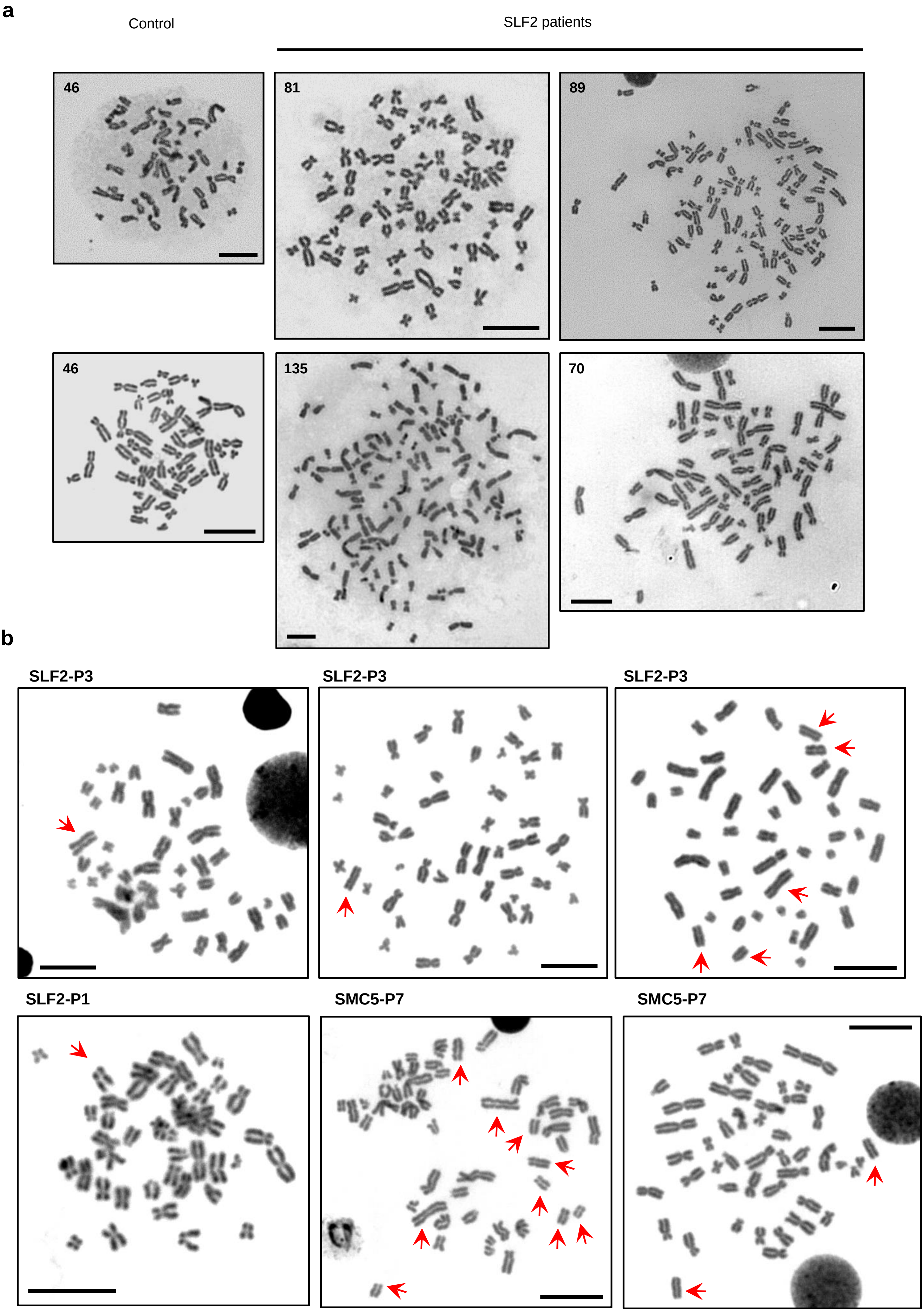
a
Control
SLF2 patients
46
81
89
46
135
70
b
SLF2-P3
SLF2-P3
SLF2-P3
SLF2-P1
SMC5-P7
SMC5-P7

Supplementary Figure 17: Representative microscopy images of SLF2 and SMC5 patient LCLs exhibiting mosaic variegated hyperploidy and sister chromatid cohesion defects

a Representative bright field microscopy images of metaphases exhibiting mosaic variegated hyperploidy derived from peripheral blood of SLF2 and SMC5 mutant patients. **b** Representative bright field microscopy images of metaphases displaying railroad chromosomes derived from peripheral blood of SLF2 and SMC5 mutant patients. In all cases, scale bars = 10 µM.

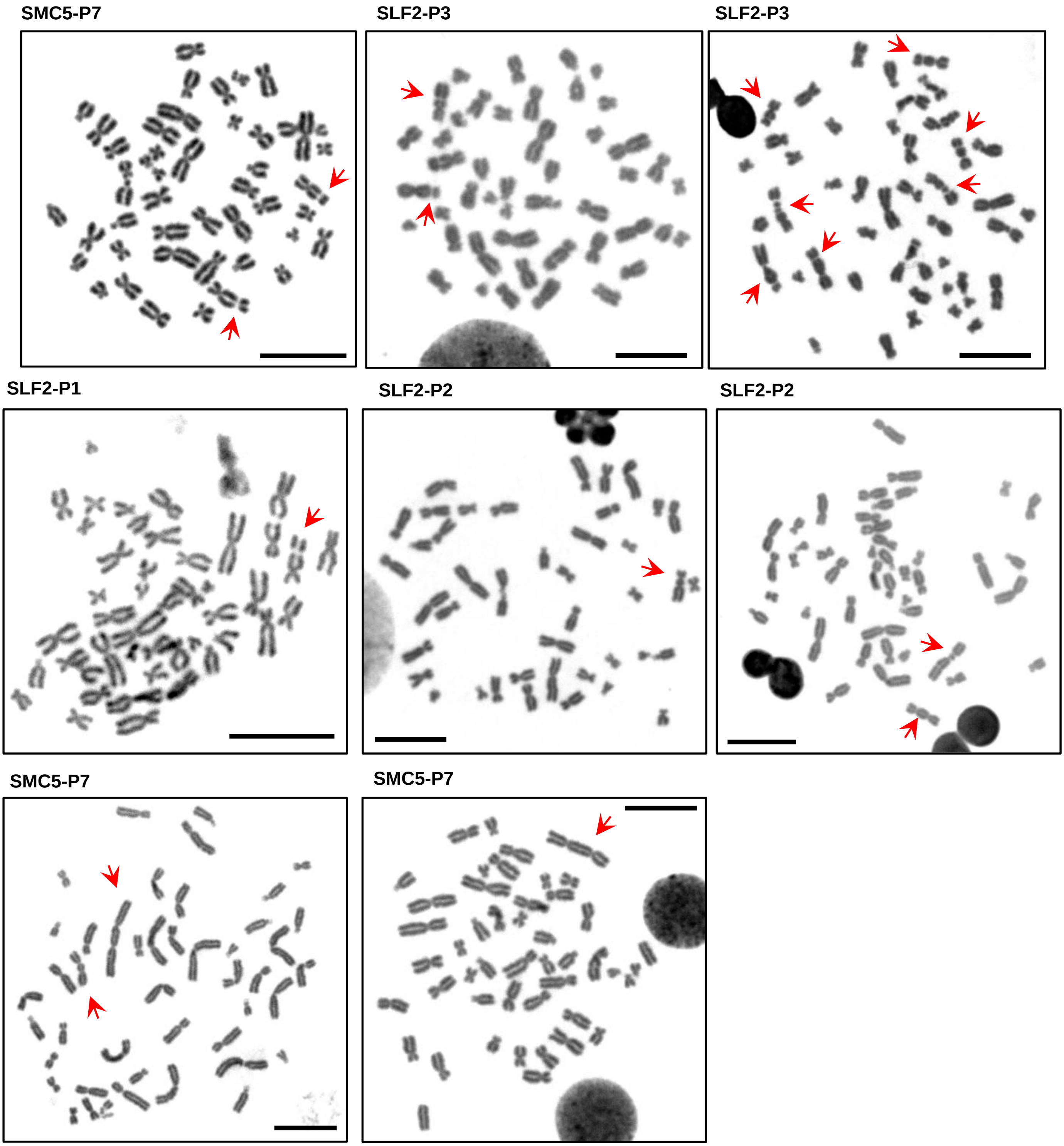

Supplementary Figure 18: SLF2 and SMC5 patient cells exhibit a unique chromosomal breakage phenotype.

Representative bright field microscopy images of metaphases displaying segmented chromosomes derived from peripheral blood of SLF2 and SMC5 mutant patients. In all cases, scale bars = 10 μM.

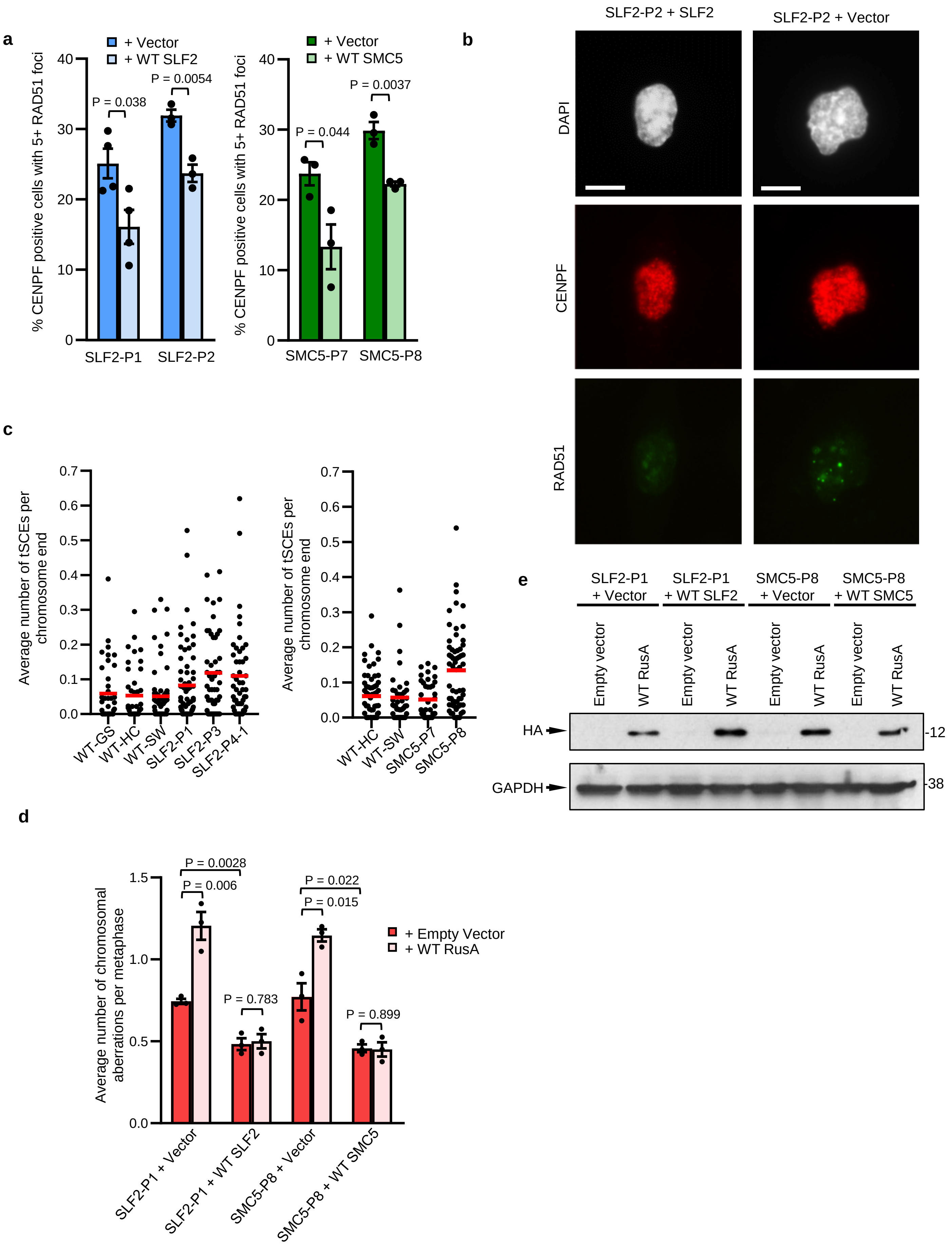
a
+ Vector
+ WT SLF2
% CENPF positive cells with 5+ RAD51 foci
P = 0.038
P = 0.0054
SLF2-P1
SLF2-P2
+ Vector
+ WT SMC5
P = 0.044
P = 0.0037
SMC5-P7
SMC5-P8
b
SLF2-P2 + SLF2
SLF2-P2 + Vector
DAPI
CENPF
RAD51
c
Average number of tSCEs per chromosome end
WT-GS
WT-HC
WT-SW
SLF2-P1
SLF2-P3
SLF2-P4-1
WT-HC
WT-SW
SMC5-P7
SMC5-P8
d
Average number of chromosomal aberrations per metaphase
P = 0.0028
P = 0.006
P = 0.022
P = 0.015
P = 0.783
P = 0.899
+ Empty Vector
+ WT RusA
SLF2-P1 + Vector
SLF2-P1 + WT SLF2
SMC5-P8 + Vector
SMC5-P8 + WT SMC5
e
SLF2-P1 + Vector
SLF2-P1 + WT SLF2
SMC5-P8 + Vector
SMC5-P8 + WT SMC5
Empty vector
WT RusA
HA
GAPDH
-12
-38

Supplementary Figure 19: SLF2 and SMC5 patient cells exhibit increased levels of recombination intermediates

a Quantification of the percentage of S/G2 (CENPF positive) cells with >5 RAD51 foci in SLF2 and SMC5 mutant fibroblast cell lines complemented with either WT SLF2, WT SMC5, or an empty vector. A minimum of 850 CENPF positive cells in total were counted over 3 independent experiments for SLF2-P2, SMC5-P7 and SMC5-P8, and 4 independent experiments for SLF2-P1. Error bars denote standard error of the mean. For statistical analysis, a Student's t-test (two-sided, equal variance) was performed. **b** Representative immunofluorescence microscopy images of cells from panel a. Scale bars = 10 µM. **c** The average number of telomeric SCEs (tSCEs) per chromosome end was quantified in WT, SLF2 and SMC5 patient-derived LCLs. The red line denotes the mean. n = 2 independent experiments. **d** Quantification of the level of chromosomal aberrations per metaphase (chromatid/chromosome gaps, breaks, fragments and chromosome radials) in complemented SLF2 and SMC5 mutant fibroblast cell lines infected with either an empty lentiviral expression vector, or a vector expressing WT RUSA. n=3 independent experiments. A minimum of 120 metaphases were counted. Error bars denote standard error of the mean. Student's t-test (two-sided, equal variance) was performed for statistical analysis. **e** Representative immunoblot analysis of HA-tagged RusA expression in SLF2 and SMC5 mutant patient fibroblasts infected with lentiviruses encoding myc-tagged WT SLF2/SMC5 or an empty vector. GAPDH was used as a loading control.

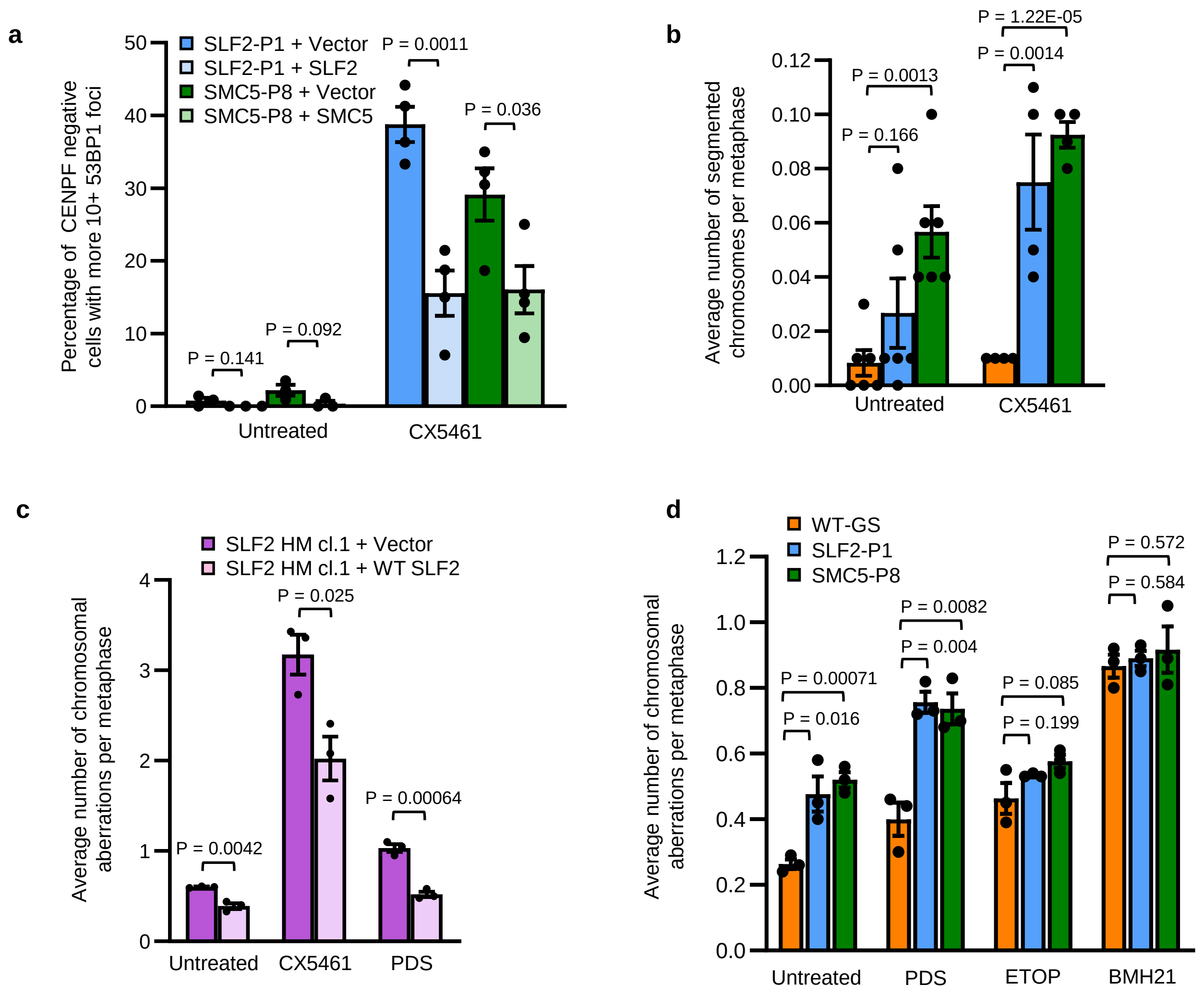

Supplementary Figure 20: SLF2 and SMC5 mutant cells exhibit increased genome instability in the presence of G-quadruplex stabilising agents

a Quantification of the percentage of G1-phase (CENPF negative) cells with >10 53BP1 bodies in SLF2 and SMC5 mutant fibroblast cell lines expressing WT SLF2, WT SMC5, or an empty vector, with or without exposure to 250 nM CX5451 for 24 h. n=4 independent experiments. A minimum of 390 G1-phase cells were counted. **b** Quantification of the average number of segmented chromosomes per metaphase in SLF2 and SMC5 patient-derived LCLs before or after exposure to 250 nM CX5461 for 24 h. n=6 independent experiments for untreated cells and n = 4 for CX5461 treated cells. A minimum of 350 metaphases were counted. **c** Quantification of the level of chromosomal aberrations (chromatid/chromosome gaps, breaks, fragments and chromosome radials) per metaphase in U-2 OS SLF2 CRISPR HM cell lines complemented with either WT SLF2 or an empty vector before or after exposure to 250 nM CX5461 or 1 µM pyridostatin (PDS) for 24 h. n=3 independent experiments. A minimum of 100 metaphases were counted. **d** Quantification of the average number of chromosomal aberrations (chromatid/chromosome gaps, breaks, fragments and radials) in metaphase spreads from SLF2 and SMC5 patient derived LCLs either left untreated or exposed to 1 µM PDS, 50 nM etoposide (ETOP) or 1 µM BMH21 for 24 h. n=3 independent experiments. A minimum of 100 metaphases were counted. In all cases, a Student's t-test (two-sided, equal variance) was performed for statistical analysis. Error bars denote SEM.

Supplementary Tables:

Table S1. Primers used for SLF2/SMC5 in vivo modelling studies.

Purpose	oligo name	Sequence
slf2 sgRNA1 CRISPR/Cas9	*slf2* sgRNA 1	5'-CAATATAGAAGAGCTGGAGG-3'
*slf2*sgRNA1 CRISPR/Cas9 efficiency	*slf2* sgRNA1 PCR primer F	5'-AAATACCCATTTTTGCCAACAG-3'
slf2 sgRNA1 CRISPR/Cas9 efficiency	*slf2* sgRNA1 PCR primer R	5'-AGGATGACAGTTTTGGCTTGTT-3'
slf2 sgRNA2 CRISPR/Cas9	*slf2* sgRNA 2	5'-TCTTATTCCAGCAGAGACCG-3'
*slf2*sgRNA2 CRISPR/Cas9 efficiency	*slf2* sgRNA2 PCR primer F	5'-TTCCTCACTCATCTCACAGACG-3'
slf2 sgRNA2 CRISPR/Cas9 efficiency	*slf2* sgRNA2 PCR primer R	5'-CCTGGACTAGTCATCGTGTTCA-3'
slf2 MO-induced suppression	*slf2* e11i11 sb MO	5'-ATGAGAAAAGTGGCTGGTATTACCT-3'
slf2 e11i11 sb MO efficiency	*slf2* e11i11 PCR primer F	5'-ACAGTGAAAGTAAAGGGGAGGAC-3'
slf2 e11i11 sb MO efficiency	*slf2* e11i11 PCR primer R	5'-AAAAGACTGATGAACGATGCCC-3'
smc5 sgRNA1 CRISPR/Cas9	*smc5* sgRNA 1	5'-GTTGCAGGTTCACGATCGGA-3'
smc5 sgRNA1 CRISPR/Cas9 efficiency	*smc5* sgRNA1 PCR primer F	5'-TGTGCTGAACATCAACCAGAG-3'
smc5 sgRNA1 CRISPR/Cas9 efficiency	*smc5* sgRNA1 PCR primer R	5'-AAACAAACGACGCTTGCATA-3'
smc5 sgRNA2 CRISPR/Cas9	*smc5* sgRNA 2	5'-AAAACATCTGTCCTGGGCCG-3'
smc5 sgRNA2 CRISPR/Cas9 efficiency	*smc5* sgRNA2 PCR primer F	5'-CAGCACGTACGATCACTCTGA-3'
smc5 sgRNA2 CRISPR/Cas9 efficiency	*smc5* sgRNA2 PCR primer R	5'-GCCAGACACAGTGGATGTGA-3'
smc5 MO-induced suppression	*smc5* e3i3 sb MO	5'-TGTAAAAACACATACTTACAGCTCT-3'
smc5 e3i3 sb MO efficiency	*smc5* e3i3 PCR primer F	5'-CCGGACCCAAACTGAACAT-3'
smc5 e3i3 sb MO efficiency	*smc5* e3i3 PCR primer R	5'-TCCTCCACTGCCTTCTGACT-3'
SMC5 mutagenesis	*SMC5*-p.R733Q-F	TGAAGAGGAAGAGCAAAAAGCAAGTACCA
SMC5 mutagenesis	*SMC5*-p.R733Q-R	5'-TGGTACTTGCTTTTTGCTCTTCCTCTTCA-3'
SMC5 mutagenesis	*SMC5*-p.H990D-F	5'-GAATTAACTCCTCATGATCAAAGTGGAGGTGAA-3'
SMC5 mutagenesis	*SMC5*-p.H990D-R	5'-TTCACCTCCACTTTGATCATGAGGAGTTAATTC-3'
SMC5 mutagenesis	*SMC5*-p.R425*-F	5'-GAAATAATTGATAAGTGAAGAGAGAGGGAAACT-3'
SMC5 mutagenesis	*SMC5*-p.R425*-R	5'-AGTTTCCCTCTCTCTTCACTTATCAATTATTTC-3'
SMC5 mutagenesis	*SMC5*-p.R372-del-F	5'-TTGACCGACAGAGGATAGGTAATACCCGC-3'
SMC5 mutagenesis	*SMC5*-p.R372-del-R	5'-GCGGGTATTACCTATCCTCTGTCGGTCAA-3'
SMC5 construct sequencing	*SMC5*-seq1	5'-GCAAGAAGACGTCAACTCCA-3'
SMC5 construct sequencing	*SMC5*-seq2	5'-CGAGCAGATAAGGTTGGGTTT-3'
SMC5 construct sequencing	*SMC5*-seq3	5'-GGAATATGAAAATGTTCGTCAGG-3'
SMC5 construct sequencing	*SMC5*-seq4	5'-TGGACGATCATATTGTACGTTTT-3'
SMC5 construct sequencing	*SMC5*-seq5	5'-CAGCAGAAGAAAAGTATGTGGTG-3'
SMC5 construct sequencing	*SMC5*-seq6	5'-ACAGTGATCTCTGAGAAGAACAAA-3
SMC5 construct sequencing	*SMC5*-seq7	5'-GCAGTGTGCTGGTGAAGTTG-3'
SLF2 construct sequencing	*SLF2*-seq1	5'-AGAAGTTGGGTGCGTGGTT-3'
SLF2 construct sequencing	*SLF2*-seq2	5'-TTTGGCTAAATATTTGGAGGCTA-3'

SLF2 construct sequencing	*SLF2*-seq3	5'-TTCCCATGAATCAGAAGAGGA-3'
SLF2 construct sequencing	*SLF2*-seq4	5'-CACTTGGAACACGGGAAAGT-3'
SLF2 construct sequencing	*SLF2*-seq5	5'-GAGCAGGAGGCTTTCCTGTA-3'
SLF2 construct sequencing	*SLF2*-seq6	5'-ATCATCCGAAACAGCCACTT-3'
SLF2 construct sequencing	*SLF2*-seq7	5'-ATGCCCAGACAGAGTTCAGG-3'
SLF2 construct sequencing	*SLF2*-seq8	5'-TTTCCTGCCATTTTCCATGT-3'
SLF2 construct sequencing	*SLF2*-seq9	5'-AGTAGGCCGACAGTTCTGGA-3'
actinb2-RT-PCR	*actinb2*_F	5'-CCACCATGTACCCTGGCATT-3'
actinb2-RT-PCR	*actinb2*_R	5'-GTCACCTTCACCGTTCCAGT-3'
slf2 mRNA expression	*slf2*-qPCR-F1	5'-TCTCCTGCAAAAGTCCAGTTC-3'
slf2 mRNA expression	*slf2*-qPCR-R1	5'-GCCTCTCAGGACTTCGTCTG-3'
slf2 mRNA expression	*slf2*-qPCR-F2	5'-ATGCGTCCTTCATCTCTGCT-3'
slf2 mRNA expression	*slf2*-qPCR-R2	5'-TCTCTGGGCTGAGGGTAAGA-3'
actinb2 mRNA expression	*actinb2*-qPCR-F	5'-TTGTTGGACGACCCAGACAT-3'
actinb2 mRNA expression	*actinb2*-qPCR-R	5'-TGAGGGTCAGGATACCTCTCTT-3'

Table S2. Primers used for sequencing of SLF2/SMC5

SLF2 Sequencing Primers	Primer name	Sequence
	SLF2-760F	5’-AAGGAGCAAATGGAGCAGAGAA-3’
	SLF2-1624F	5’-TGCGCTCAGAATATGGCACT-3’
	SLF2-2556F	5’-GTCTGATGTAGCAGCTGTGTT-3’,
	SLF2-2961F	5’-TGAACTCTCCAGTCATCCCCA-3’
	SLF2-1768R	5’-GGCTTTATCTGAAGGTGCTGC-3’
	SLF2-2575R	5’-ACACAGCTGCTACATCAGACA-3’
	SLF2-3437R	5’-CTGGCGACCAAGTCTTTCAC-3’
**SMC5 Sequencing Primers**	**Primer name**	**Sequence**
	SMC5-300F	5’-ACCTGCTTTCATGGGACGAG-3’
	SMC5-975F	5’-AGAAAAGGCAACAGATATTAAGGAG-3’
	SMC5-1563F	5’-GGTTTTCCTCAAAGAGGTTCGTG-3’
	SMC5-1681F	5’-GTTTTCCTCAAAGAGGTTCGTG-3’
	SMC5-2188F	5’-GAGGAAGAGCGAAAAGCAAGT-3’
	SMC5-2322F	5’-TGCTTTTCGCTCTTCCTCTTCA-3’
	SMC5-2486F	5’-CCGCATCTTCACAACTCCGT-3’
	SMC5-687R	5’-GCATGAGGTCTCGAGCTGTTT-3’
	SMC5-1194R	5’-GGGCTGAAGATTCTCGCAGT-3’
	SMC5-1234R	5’-TTCTCCTCTGTCGGTCAAGC-3’
	SMC5-3178R	5’-TTTTGCAGGAGCTTTGGTGT-3’

Table S3. Primers used for the generation of SLF2 and SMC5 deletion/mutation constructs

**SLF2 deletion constructs**	**Sequences of primer pairs**
SLF2 Δ1	5’-TCCAGCACAGTGGCGGCCGCAACAGCTCCAGAAGCCTTAG-3’ and 5’-CTAAGGCTTCTGGAGCTGTTGCGGCCGCCACTGTGCTGGA-3’
SLF2 Δ2	5’-AGAAGAATGATAGAGATCGAAATTCTGGCAATTCTGGCCA-3’ and 5’-TGGCCAGAATTGCCAGAATTTCGATCTCTATCATTCTTCT-3’
SLF2 Δ3	5’-GTGATGTGTTGCGCTTAGAAAACCTAGACAGTGATGAGGA-3’ and 5’-TCCTCATCACTGTCTAGGTTTTCTAAGCGCAACACATCAC-3’
SLF2 Δ4	5’-CAGGAAATTCCAATGCAGGTCTGTTTCGGATGATGTCAGT-3’ and 5’-ACTGACATCATCCGAAACAGACCTGCATTGGAATTTCCTG-3’
SLF2 Δ5	5’-AGATTTTTTTGACAACACAAAGGCAACTGAGACAGTGCCT-3’ and 5’-AGGCACTGTCTCAGTTGCCTTTGTGTTGTCAAAAAAATCT-3’
SLF2 Δ6:	5’-TGGGCATAAATGAACTCTCCTAGGGGCCCGTTTAAACCCG-3’ and 5’-CGGGTTTAAACGGGCCCCTAGGAGAGTTCATTTATGCCCA-3’
SLF2 Δ7	5’-GCCGAGGCATTAAATCCCCACCTGTCCCTGTGTTAAAGTG-3’ and 5’-CACTTTAACACAGGGACAGGTGGGGATTTAATGCCTCGGC-3’
SLF2 Δ8	5’-CGTCTGCTTATCACTATGTCCCAATTTTTTCAACACTTCC-3’ and 5’-GGAAGTGTTGAAAAAATTGGGACATAGTGATAAGCAGACG-3’
SLF2 Δ9	5’-GGAAAGAAAGTGAAGATTCACAGCTGGTCCCTAATTGGAC-3’ and 5’-GTCCAATTAGGGACCAGCTGTGAATCTTCACTTTCTTTCC-3’
SLF2 Δ10	5’-ACAACCTCCTGTGGTTGGTATGTTCTCATTCTTTTTCTTC-3’ and 5’-GAAGAAAAAGAATGAGAACATACCAACCACAGGAGGTTGT-3’
SLF2 Δ11	5’-CACTGAAAAGAAAACTAAGGTCCCCAATCAGAATTGGAGA-3’ and 5’-TCTCCAATTCTGATTGGGGACCTTAGTTTTCTTTTCAGTG-3’.
MBR1	5’-GAACATGCGGCCGCTTCAATCAGTATACCTTG-3’ and 5’-CGCTCTAGAGCCTAACTAACTTCACCGACTAA-3’
MBR2	5’-GAACATGCGGCCGCTTCAATCAGTATACCTTG-3’ and 5’-CAGCATTCTAGACGCTAAGAATCTGGTACCCA-3’.
**SLF2 and SMC5 mutant constructs**	**Sequences of primer pairs**
SLF2 p.Ser815Ter	5’-TGTTTCGGATGATGTGAGTTCATACAGACTG-3’ and 5’-CAGTCTGTATGAACTCACATCATCCGAAACA-3’
SLF2 p.Arg336Lysfs:	5’-AATTCCCTGAAAAAAAGAAAAAGGAACTCTG-3’ and 5’-CAGAGTTCCTTTTTCTTTTTTTCAGGGAATT-3’
SLF2 p.ΔSer907Phefs	5’-TCCTGAAACCAACATTTTAAATG-3’ and 5’-AAAAAATTGGCTTATAAGATGAATC—3’
SLF2 p.Asn861Ile	5’-GTGTTTTTCATTATGGGGATTGATTTTAG-3’ and 5’-AGCTGCTACATCAGACAATG-3’
SLF2 .ΔAla1085_Arg1110	5’-AAACACTTTGTGCTACTC-3’ and 5’-CTGCTTTTCAAGTTCTAAATG-3’
SLF2 p.Aps783Serfs	5’-TCAGATTTTTTTGACAACAC-3’ and 5’-TGTTTTTCCCGATTTAAGAATAAG-3’
SMC5 p.Arg425Ter	5’-AATTGATAAGGGAAGAGAGAGG-3’ and 5’-ATTTCGCCTTCACATAATG-3’
SMC5 p.ΔArg372	5’-GAGAATAGGTAATACCCGC-3’ and 5’-TGTCGGTCAAGCTCTTCA-3’
SMC5 p.His990Asp	5’-AACTCCTCATGATCAAAGTGG-3’ and 5’-AATTCATGCAGTTGAGTAC-3’

Table S4. Primers used for RT-PCR expression studies

Experiment	Primer name	Identifier	Sequence
**Expression studies**			
RT-PCR	SLF2_ex16_common_F	P1	GTGCAGATGAAGCCTTCTGA
RT-PCR	SLF2_1173_ex20_R	P2	GGTACCCAGAAGTCATGAAGC
RT-PCR	SLF2_1186_ex19_R	P3	TGAAGAGTGCCATTCAGCAA
RT-PCR	SLF2-E1-FOR	P4	CGCGCTGCCATCTGAGACCC
RT-PCR	SLF2-E3-REV	P5	GGACAGGCTGCTCCTGCTGC
RT-PCR	SLF2-E14-FOR	P6	GGACAGGCTGCTCCTGCTGC
RT-PCR	SLF2-I19-REV	P7	GGTGCCTGAACTCTGTCTGGGC
RT-PCR	SLF2-E20-REV	P8	TGAAGAGTGCCATTCAGCAAAACT
