# Supplementary material for: Pathogenic variants in SLF2 and SMC5 cause segmented chromosomes and mosaic variegated hyperploidy
Source: Nat Commun. 2022 Nov 4;13:6664. doi: 10.1038/s41467-022-34349-8 (PMC9636423; doi:10.1038/s41467-022-34349-8)
Supplement: Supplementary file 3 — Description of Additional Supplementary Files [file 41467_2022_34349_MOESM3_ESM.pdf]

Description of Additional Supplementary Files

File Name: Supplementary Data 1
Description: Table listing clinical phenotype data of individuals with *SLF2* and *SMC5* mutations. Hom, homozygous; Het, heterozygous; y, year; m, month; OFC; occipital frontal circumference; Wgt, weight; Lgth, length; Hgt, height; SD, standard deviation; ND; not done; IUGR, intrauterine growth restriction.

File Name: Supplementary Data 2
Description: Table listing variants identified by whole exome sequencing in patient SLF2-P1, assuming an autosomal recessive mode of inheritance. Chr, chromosome; SNV, single nucleotide variant; HOM, homozygous.

File Name: Supplementary Data 3
Description: Table listing variants identified by whole exome sequencing in patient SLF2-P2, assuming an autosomal recessive mode of inheritance. HOM, homozygous; HET, heterozygous.

File Name: Supplementary Data 4
Description: Table listing variants identified by whole exome sequencing in patient SLF2-P3, assuming an autosomal recessive mode of inheritance. Chr, chromosome; HOM, homozygous; HET, heterozygous.

File Name: Supplementary Data 5
Description: Table listing variants identified by whole exome sequencing in patients SLF2-P4-1 and SLF2-P4-2, assuming an autosomal recessive mode of inheritance. HOM, homozygous; HET, heterozygous.

File Name: Supplementary Data 6
Description: Table listing variants identified by whole exome sequencing in patient SLF2-P5, assuming an autosomal recessive mode of inheritance. HOM, homozygous.

File Name: Supplementary Data 7
Description: Table listing variants identified by whole exome sequencing in patient SLF2-P6, assuming an autosomal recessive mode of inheritance. HOM, homozygous; HEM, hemizygous.

File Name: Supplementary Data 8
Description: Table listing variants identified by whole exome sequencing in patient SMC5-P7, assuming an autosomal recessive mode of inheritance. Chr, chromosome; HET, heterozygous.

File Name: Supplementary Data 9
Description: Table listing variants identified by whole exome sequencing in patient SMC5-P8, assuming an autosomal recessive mode of inheritance. SNV, single nucleotide variant.

File Name: Supplementary Data 10
Description: Table listing variants identified by whole exome sequencing in patients SMC5-P9-1 and P9-2, assuming an autosomal recessive mode of inheritance. SNV, single nucleotide variant.
