# Supplementary material for: Pathogenic variants in SLF2 and SMC5 cause segmented chromosomes and mosaic variegated hyperploidy
Source: Nat Commun. 2022 Nov 4;13:6664. doi: 10.1038/s41467-022-34349-8 (PMC9636423; doi:10.1038/s41467-022-34349-8)
Supplement: Supplementary file 14 — Reporting Summary [file 41467_2022_34349_MOESM14_ESM.pdf]

nature portfolio

Corresponding author(s): Grant S. Stewart, Erica E. Davis, Cedric Le Caignec

Last updated by author(s): 19/10/2022

Reporting Summary

Nature Portfolio wishes to improve the reproducibility of the work that we publish. This form provides structure for consistency and transparency in reporting. For further information on Nature Portfolio policies, see our Editorial Policies and the Editorial Policy Checklist.

Statistics

For all statistical analyses, confirm that the following items are present in the figure legend, table legend, main text, or Methods section.

n/a	Confirmed	
☐	☒	The exact sample size (*n*) for each experimental group/condition, given as a discrete number and unit of measurement
☐	☒	A statement on whether measurements were taken from distinct samples or whether the same sample was measured repeatedly
☐	☒	The statistical test(s) used AND whether they are one- or two-sided<br>*Only common tests should be described solely by name; describe more complex techniques in the Methods section.*
☒	☐	A description of all covariates tested
☒	☐	A description of any assumptions or corrections, such as tests of normality and adjustment for multiple comparisons
☐	☒	A full description of the statistical parameters including central tendency (e.g. means) or other basic estimates (e.g. regression coefficient) AND variation (e.g. standard deviation) or associated estimates of uncertainty (e.g. confidence intervals)
☐	☒	For null hypothesis testing, the test statistic (e.g. *F*, *t*, *r*) with confidence intervals, effect sizes, degrees of freedom and *P* value noted<br>*Give P values as exact values whenever suitable.*
☒	☐	For Bayesian analysis, information on the choice of priors and Markov chain Monte Carlo settings
☒	☐	For hierarchical and complex designs, identification of the appropriate level for tests and full reporting of outcomes
☒	☐	Estimates of effect sizes (e.g. Cohen's *d*, Pearson's *r*), indicating how they were calculated

Our web collection on statistics for biologists contains articles on many of the points above.

Software and code

Policy information about availability of computer code

Data collection	Nikon NIS Elements software (https://www.microscope.healthcare.nikon.com/products/software/nis-elements/viewer) and ZenPro software (https://www.zeiss.com/microscopy/en/products/software/zeiss-zen-lite.html) was used to obtain immunofluorescence microscopy images. Vertebrate Automated Screening Technology (VAST) Bioimager (Union Biometrica) was used to capture images of zebrafish embryos (https://www.unionbio.com/vast/).
Data analysis	We have used AlphaFold (https://alphafold.ebi.ac.uk/), PyMOL (https://pymol.org/2/) and DynaMut (http://biosig.unimelb.edu.au/dynamut) for structural analysis of SMC5 variants. GraphPad Prism version 9.4.1 was used to generate histograms and dot plots and calculate statistical significance. CHOPCHOP (version 2) was used to identify sgRNA sequences (https://chopchop.cbu.uib.no/). ImageJ (version 1.8.0_172) was used to measure lateral head size, ceratohyal angle zebrafish embryos (https://imagej.nih.gov/ij/). An ICTN plugin for ImageJ was used to quantify nuclei (https://bioimage.ucsb.edu/docs/automatic-nuclei-counter-plugin-imagej). Gene Tools was used design morpholinos to slf2 and smc5 (https://www.gene-tools.com/). Bioinformatic predictions of gene variants that affect splicing were carried out using Human Splicing Finder (http://www.umd.be/HSF3/HSF/), MaxEntScan (http://genes.mit.edu/burgelab/maxent/Xmaxentscan_scoreseq/), NNSPLICE (https://www.fruitfly.org/seq_tools/splice.html) and Alamut (https://www.sophiagenetics.com/platform/alamut-visual-plus/). Clustal Omega was used to align protein sequences (https://www.ebi.ac.uk/Tools/msa/clustalo/). Gene variant pathogenicity was predicted using Polyphen-2 (http://genetics.bwh.harvard.edu/pph2/).

For manuscripts utilizing custom algorithms or software that are central to the research but not yet described in published literature, software must be made available to editors and reviewers. We strongly encourage code deposition in a community repository (e.g. GitHub). See the Nature Portfolio guidelines for submitting code & software for further information.

Data

Policy information about availability of data

All manuscripts must include a data availability statement. This statement should provide the following information, where applicable:

- Accession codes, unique identifiers, or web links for publicly available datasets
- A description of any restrictions on data availability
- For clinical datasets or third party data, please ensure that the statement adheres to our policy

The datasets generated during WES that support this study are available from the corresponding authors upon reasonable request. Informed consents from patients do not cover the deposition of sequencing data from the patient samples, but data can be shared for research purposes with permission of the patient or his/her legal guardian. Gene variant frequency was obtained from the gnomAD database (https://gnomad.broadinstitute.org/). Accession codes for genes/proteins analysed within this study are: Human SLF2 (NM_018121.4, NM_001136123.2, NP_060591.3), Human SMC5 (NM_015110.4, NP_055925.2), zebrafish slf2 (XM_002664123.6, XP_002664169.3), zebrafish smc5 (NM_001193541.1, NP_001180470.1). Plasmids obtained from Addgene (https://www.addgene.org/) used in this study: pLV-hTERT-IRES-hygro (Addgene #85140), psPax2 (Addgene #12260) and pMD2.G (Addgene #12259). PDB files used within this study to model the structural impact of SMC5 patient variants: Saccharomyces cerevisiae Smc5 (PDB: 3HTK), Pyrococcus furiosus RAD50 (PDB: 1F2T and 1FTU). AlphaFold models used to facilitate structural predictions: human SMC5 (AF-Q8IY18-F1).

Field-specific reporting

Please select the one below that is the best fit for your research. If you are not sure, read the appropriate sections before making your selection.

☒ Life sciences ☐ Behavioural & social sciences ☐ Ecological, evolutionary & environmental sciences

For a reference copy of the document with all sections, see nature.com/documents/nr-reporting-summary-flat.pdf

Life sciences study design

All studies must disclose on these points even when the disclosure is negative.

Sample size	No statical tests were performed to determine sample size. Sample size for each experiment is indicated in the legend. Sample size was based on previous experimental experience using similar studies and assays employed within the field e.g. Abu-Libdeh et al (2022) J Clin Invest. 132:e147301; Bayley et al (2022) Mol Cell. 82:1924-1939; Higgs et al (2018) Mol Cell. 71:25-41; Reynolds et al (2017) Nature Genet. 49:537-549; Higgs et al (2015) Mol Cell. 59:462-77. The sample size of each experiment, such as how many cells were analyzed for each independent biological replicate, is indicated in the relevant Figure Legends.
Data exclusions	No data was excluded from the analysis.
Replication	Each experiment was repeated multiple times, with the number of independent experiments (at least 3) being stated in the relevant Figure Legends.
Randomization	All samples used in this study, including cultured cells, were allocated randomly to each condition.
Blinding	Samples in each experiment to be analyzed by microscopy were randomized so the investigator was counting the experiment blind. Samples for gene sequencing, immunoblotting, immunoprecipitation, or samples that required the addition of different genotoxic compounds were known to the experimenter when preparing samples. In most cases where the identity of each sample was known to the experimenter was due to the fact that much of the data presented within the manuscript was provided back to the referring clinician as part of a report to feedback to the affected family/patient about our work confirming the pathogenicity of the gene variants identified by whole exome sequencing. This is essential to reduce the possibility of mixing up patient samples or patient results that could compromise the clinical report.

Reporting for specific materials, systems and methods

We require information from authors about some types of materials, experimental systems and methods used in many studies. Here, indicate whether each material, system or method listed is relevant to your study. If you are not sure if a list item applies to your research, read the appropriate section before selecting a response.

Materials & experimental systems

n/a	Involved in the study
☐	☒ Antibodies
☐	☒ Eukaryotic cell lines
☒	☐ Palaeontology and archaeology
☐	☒ Animals and other organisms
☐	☒ Human research participants
☒	☐ Clinical data
☒	☐ Dual use research of concern

Methods

n/a	Involved in the study
☒	☐ ChIP-seq
☒	☐ Flow cytometry
☒	☐ MRI-based neuroimaging

Antibodies

Antibodies used

RAD18 (Fortis Life Sciences, A301-340A), SMC5 (Fortis Life Sciences, A300-236A), SMC6 (Fortis Life Sciences, A300-237A), SLF2 (generated in house), GAPDH (Genetex, GTX100118), Myc (Abcam, ab32), GFP (SCBT, sc-9996), HA (SCBT, sc-7392), ATR (Fortis Life Sciences, A300-137A), phospho-ATR (Thr1989) (GeneTex, GTX128145;1:500), FANCD2 (SCBT, sc-20022), CHK1 (SCBT, sc-8408), phospho-CHK1 (Ser345) (Cell Signaling Technology, 234), NBS1 (Genetex, GTX70224); phospho-NBS1 (Ser343) (Abcam, 47272); SMC1 (Fortis Life Sciences, A300-055A); phospho-SMC1 (Ser966) (Fortis Life Sciences, A300-050A), α-Tubulin (Sigma-Aldrich, T9026), anti-γH2AX(pS139) (Sigma-Aldrich, 05-636) and anti-GFP (PABG1, Chromotek), anti-pHH3 (SCBT, sc-374669), CENPA (Abcam, Ab13939), 53BP1 (Novus Biologicals, NB100-304), CENPF/Mitosin (Abcam, Ab5), CENPF/Mitosin (BD Transduction Laboratories, 610768), α-Tubulin (Sigma-Aldrich, B-5-1-2), PCNT (Abcam, Ab4448), and RAD51 (Sigma-Aldrich, PC130), anti-BrdU antibody (clone BU1/75, ICR1; Abcam, ab6326) and mouse anti-BrdU antibody (clone B44; BD Biosciences, 347583), anti-rabbit IgG Alexa Fluor 488 (Thermo Fisher Scientific, A11070) and anti-mouse IgG Alexa Fluor 594 (Thermo Fisher Scientific, A11032)

Validation

All commercially available antibodies were used as per the manufacturer's guidelines and used for the techniques in which they have been validated by the manufacturers. The SLF2 antibody generated in house was validated in Raschle, M. et al. 2015; Science.

The antibodies available from Fortis Life Sciences have all been validated for Western blotting and/or immunoprecipitation:
RAD18: https://www.fortislife.com/products/primary-antibodies/rabbit-anti-rad18-antibody/BETHYL-A301-340
SMC5: https://www.fortislife.com/products/primary-antibodies/rabbit-anti-smc5-antibody/BETHYL-A300-236
SMC6: https://www.fortislife.com/products/primary-antibodies/rabbit-anti-smc6-antibody-affinity-purified/BETHYL-A300-237
ATR: https://www.fortislife.com/products/primary-antibodies/rabbit-anti-atr-antibody/BETHYL-A300-137
SMC1: https://www.fortislife.com/products/primary-antibodies/rabbit-anti-smc1-antibody/BETHYL-A300-055
Phospho-SMC1: https://www.fortislife.com/products/primary-antibodies/rabbit-anti-phospho-smc1-s966-antibody/BETHYL-A300-050

Antibodies to epitope tags have been verified for Western blotting by the manufacturer:
Myc: https://www.abcam.com/myc-tag-antibody-9e10-ab32.html
GFP: https://www.scbt.com/p/gfp-antibody-b-2?requestFrom=search
GFP: https://www.ptglab.com/products/GFP-antibody-rabbit-polyclonal-PABG1.htm
HA: https://www.scbt.com/p/ha-probe-antibody-f-7?requestFrom=search

We have previously validated these antibodies: anti-ATR, anti-phospho-ATR, anti-FANCD2, anti-Chk1, anti-phospho-Chk1, anti-Nbs1, anti-phospho-Nbs1, anti-SMC1, anti-phospho-SMC1, anti-γH2AX, anti-53BP1, anti-CENPA, anti-Rad51, anti-CENPF/Mitosin, anti-pHH3, anti-α-Tubulin, anti-PCNT and anti-BrdU antibodies, for Western blotting and immunofluorescence in the following publications:

Abu-Libdeh B, Jhujh SS, Dhar S, Sommers JA, Datta A, Longo GMC, Grange LJ, Reynolds JJ, Cooke SL, McNee GS, Hollingworth R, Woodward BL, Ganesh AN, Smerdon SJ, Nicolae CM, Durlacher-Betzer K, Molho-Pessach V, Abu-Libdeh A, Meiner V, Moldovan G-L, Roukos V, Harel T, Brosh Jr. RM, Stewart GS. (2022). RECON Syndrome is a genome instability disorder caused by mutations in the DNA helicase RECQL1. J Clin Invest. 132:e147301

Bayley R, Borel V, Moss RJ, Sweatman E, Ruis P, Ormrod A, Goula A, Mottram RMA, Stanage T, Hewitt G, Saponaro M, Stewart GS, Boulton SJ, Higgs MR. (2022). H3K4 methylation by SETD1A/BOD1L facilitates RIF1-dependent NHEJ. Mol Cell. 82:1924-1939

Higgs MR, Sato K, Reynolds JJ, Begum S, Bayley R, Goula A, Vernet A, Paquin KL, Skalnik DG, Kobayashi W, Takata M, Howlett NG, Kurumizaka H, Kimura H, Stewart GS. (2018). Histone methylation by SETD1A protects nascent DNA through the nucleosome chaperone activity of FANCD2. Mol Cell. 71:25-41

Reynolds JJ, Bicknell LS, Carroll P, Higgs MR, Shaheen R, Murray JE, Papadopoulos DK, Leitch A, Murina O, Tarnauskaitė Ž, Wessel SR, Zlatanou A, Vernet A, Kriegsheim A, Mottram RMA, Logan CV, Bye H, Li Y, Brean A, Maddirevula S, Challis RC, Skouloudaki K, Almoisheer A, Alsaif HS, Amar A, Prescott NJ, Bober MB, Duker A, Faqeih E, Seidahmed MZ, Tala SA, Alswaid A, Ahmed S, Al-Aama JY, Altmüller J, Balwi MA, Brady AF, Chessa L, Cox H, Fischetto R, Heller R, Henderson BD, Hobson E, Nürnberg P, Percin EF, Peron S, Spaccini L, Quigley AJ, Thakur S, Wise CA, Yoon G, Alnemer M, Tomancak P, Yigit G, Taylor AMR, Reijns MAM, Simpson MA, Cortez D, Alkuraya FS, Mathew CG, Jackson AP, Stewart GS. (2017). Mutations in DONSON disrupt replication fork stability and cause microcephalic dwarfism. Nature Genet. 49:537-549

Higgs MR, Reynolds JJ, Winczura A, Blackford AN, Borel V, Miller ES, Zlatanou A, Nieminuszczy J, Ryan EL, Davies NJ, Stankovic T, Boulton SJ, Niedzwiedz W, Stewart GS. (2015). BOD1L Is Required to Suppress Deleterious Resection of Stressed Replication Forks.

	Mol Cell. 59:462-77.

Eukaryotic cell lines

Policy information about cell lines

Cell line source(s)	Patient-derived lymphoblastoid cell lines (LCLs) were generated from peripheral blood samples with Epstein Barr virus (EBV) transformation using standard methods. Dermal primary fibroblasts were grown from skin-punch biopsies and were immortalized with a lentivirus expressing human telomerase reverse transcriptase (hTERT). U-2 OS cells were obtained from ATCC and 293FT cells were purchased from Invitrogen.
Authentication	All patient derived cell lines were authenticated by sequencing and verifying the presence of the relevant mutations.
Mycoplasma contamination	All cell lines were routinely tested for, and confirmed to be negative for, mycoplasma contamination.
Commonly misidentified lines (See ICLAC register)	No commonly misidentified cell lines were used in this study.

Animals and other organisms

Policy information about studies involving animals; ARRIVE guidelines recommended for reporting animal research

Laboratory animals	Wild type (WT: ZDR or NIH) zebrafish adults or transgenic -1.4col1a1:egfp18 zebrafish adults were maintained on an AB background and subjected to natural matings to generate embryos for microinjection and/or phenotyping. Zebrafish sex is unknown until animals are ~3 months old. Thus, in the larvae at <5days post fertilization, it is not possible to know how many males and females were included, and there should be no sex-dependent effects at this stage. However, adults that were used to generate embryos were crossed in a 1 male to 1 female ratio.
Wild animals	No wild animals were used in this stusy.
Field-collected samples	No field collected samples were used.
Ethics oversight	All zebrafish experiments were performed according to protocols approved by the Duke University and Northwestern University institutional animal care and use committees (IACUC).

Note that full information on the approval of the study protocol must also be provided in the manuscript.

Human research participants

Policy information about studies involving human research participants

Population characteristics	The characteristics and clinical phenotypes of patients with mutations in SLF2 and SMC5 are included in Supplementary Table 1.
Recruitment	Patients with microcephaly and short stature who possessed mutations in SLF2 and SMC5 were identified by individual groups/clinicians using whole exome sequencing, and the collaboration to study the pathological significance of the identified SLF2 and SMC5 mutations was established via GeneMatcher. Patients were only selected to be recruited into the study if they were identified to have inherited biallelic, potentially pathogenic variants in either the SLF2 or SMC5 genes that were inherited from both parents and segregated with the disease. Any patients with variants in either SLF2 or SMC5 that were demonstrated not to be pathogenic (i.e. the variants did not affect mRNA expression, gene splicing, protein stability, protein localisation) or that re-expression of the WT gene in patient-derived cell lines did not complement any identified cellular defects, were excluded from the study. Patients with mono-allelic or de novo variants in either SLF2 or SMC5 were excluded from the study due to difficulties with definitively demonstrating the pathogenicity of these variants in a small patient cohort.
Ethics oversight	Informed consent was obtained from all participating families to take clinical samples and to publish clinical information in accordance with local approval regulations. This study was approved by the West Midlands, Coventry and Warwickshire Research Ethics Committee (REC: 20/WM/0098), the Scottish Multicentre Research Ethics Committee (REC: 05/MRE00/74) and the Institutional Review Boards of Yokohama City University Graduate School of Medicine (ID: A190800001) and Jichi Medical University (ID: G21-V06).

Note that full information on the approval of the study protocol must also be provided in the manuscript.
